# A Computational Framework for Bioimaging Simulation

**DOI:** 10.1371/journal.pone.0130089

**Published:** 2015-07-06

**Authors:** Masaki Watabe, Satya N. V. Arjunan, Seiya Fukushima, Kazunari Iwamoto, Jun Kozuka, Satomi Matsuoka, Yuki Shindo, Masahiro Ueda, Koichi Takahashi

**Affiliations:** 1 Laboratory for Biochemical Simulation, Quantitative Biology Center, RIKEN, Suita, Osaka, Japan; 2 Laboratory for Cell Signaling Dynamics, Quantitative Biology Center, RIKEN, Suita, Osaka, Japan; 3 Institute for Advanced Biosciences, Keio University, Tsuruoka, Yamagata, Japan; 4 Graduate School of Frontier Biosciences, Osaka University, Suita, Osaka, Japan; University of Pécs Medical School, HUNGARY

## Abstract

Using bioimaging technology, biologists have attempted to identify and document analytical interpretations that underlie biological phenomena in biological cells. Theoretical biology aims at distilling those interpretations into knowledge in the mathematical form of biochemical reaction networks and understanding how higher level functions emerge from the combined action of biomolecules. However, there still remain formidable challenges in bridging the gap between bioimaging and mathematical modeling. Generally, measurements using fluorescence microscopy systems are influenced by systematic effects that arise from stochastic nature of biological cells, the imaging apparatus, and optical physics. Such systematic effects are always present in all bioimaging systems and hinder quantitative comparison between the cell model and bioimages. Computational tools for such a comparison are still unavailable. Thus, in this work, we present a computational framework for handling the parameters of the cell models and the optical physics governing bioimaging systems. Simulation using this framework can generate digital images of cell simulation results after accounting for the systematic effects. We then demonstrate that such a framework enables comparison at the level of photon-counting units.

## Introduction

All scientific measurements are subject to some uncertainties. Experimental accuracy and precision must be always estimated to establish the validity of our results [1, 2]. It is also true for measurements using bioimaging techniques such as fluorescence microscopy. The measurements are generally influenced by systematic effects that arise from the stochastic nature of biological cells, the imaging apparatus, and optical physics. Such systematic effects are always present in all bioimaging systems and hinder the validation of the mathematical models of biological cells. For example, the local precision of reconstructed images obtained by precise localization microscopy, such as stochastic optical reconstruction microscopy (STORM), and photoactivated localization microscopy (PALM) is particularly limited by the systematic effects that are governed by camera specifications and its operating conditions [3–5]. The limitation constrains the validation of the mathematical models of the biological dynamics.

Theory of model validation is often applied to obtain valid mathematical models in physics and engineering fields [6–8]. It can be also applied to biological science, because it offers a formal representation of the progressive build-up of trust in the mathematical model of interest. In a standard exercise of model validation, one performs an experiment and in parallel, runs a simulation of the model. Then, using metrics controlled by the parameters embedded in the model and the experimental configuration, the output of the model simulation is iteratively compared and analyzed with the actual experimental output. There are three important parts in the iterative process. (1) The experimental outputs are generally influenced by the systematic effects that arise from various sources in the bioimaging process. The outputs of the model simulation are usually not presented in the most efficient way for comparison with the experimental outputs. Simulations of the experimental techniques and their operating conditions are essential for proper comparison and analysis. (2) The predictive capability of the model is to go beyond the well-known parameter domain and into a new parameter domain of unknown conditions and outcome. Calibration and validation are one of the important processes of parameter adjustment in each domain. Calibration is defined as the process of improving the agreement of a set of simulated outputs with a set of actual outputs obtained under well-controlled experimental systems. Validation is defined as the process of quantifying our confidence in the predictive capability for a given application. (3) Analyses of parameter sensitivity and limitation are also important to reduce the size of the parameter domain.

In this article, we focus on the first (comparison) issue/part. In order to properly compare spatial models of biological cell with actual cell images, we propose a computational framework for managing parameter dependences by defining a uniform interface and common organizational principles governing the systematic effects. Such a framework allows us to efficiently handle the parameters defined in a spatial cell model and the physical principles governing the bioimaging techniques and their operating conditions. Using this framework, we program bioimaging simulation modules to generate digital images of the cell simulation results after accounting for the systematic effects. The intensity of the simulated images corresponds to the number of photons detected in a light-sensitive device. Thus, the framework streamlines the comparison at the level of photon-counting units. In particular, we implement the simulation modules for relatively simple microscopy systems: total internal reflection fluorescence microscopy (TIRFM) and laser-scanning confocal microscopy (LSCM). We then evaluate the performance of the simulation modules by comparing a simulated image with an actual image for simple particle models of fluorescent molecules. Thus, these images are comparable at the level of photon-counting units. Each simulated image is visually similar to the corresponding real one. In addition, using the LSCM simulation module, we compared a more complex cell model with real cell images obtained by the actual LSCM system. We construct the following spatial cell models for the comparison: (i) the ERK nuclear translocation model for the epidermal growth factor (EGF) signaling pathway, and (ii) the self-organizing wave model of phosphatase and a tensinin homolog (PTEN) for the chemotactic pathway of *Dictyostelium discoideum*. Using a test version of the TIRFM simulation module, we compared the oscillation model of the Min proteins of *Escherichia coli* with actual cell images [9].

## Methods

### Computational framework

**Fig 1:**
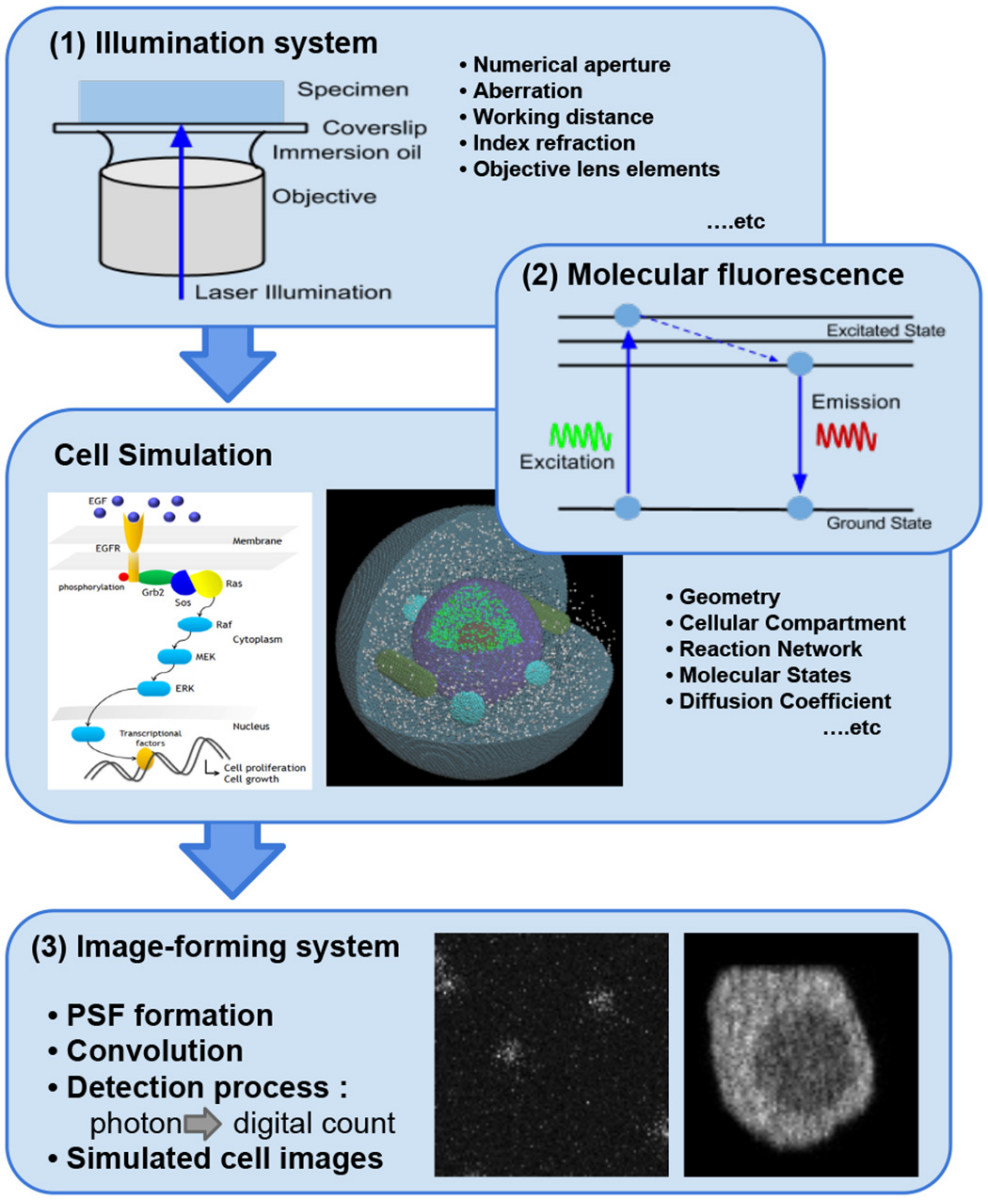
Schematic overview of the computational framework. Direction of photon propagation is presented by thick blue arrows.

To render the simulated output of a spatial cell model well suited for comparison at the level of photon-counting units, we propose a computational framework for simulating the passage of photons through fluorescent molecules and the optical system. Simulations using this framework can generate simulated digital images after accounting for the systematic effects that are governed by the parameters embedded in spatial cell model and optics system. An overview of the computational framework is schematically shown in Fig 1. The simulation of the optical system is composed of three components: (1) an illumination system, (2) molecular fluorescence, and (3) an image-forming system. The illumination system transfers photon flux from a light source to the spatial cell model to create a prescribed photon distribution and maximize the photon flux delivered to the cell model. Fluorophores defined in the cell model absorb photons from the distribution and are quantum mechanically excited to higher energy states. Molecular fluorescence is the result of physical and chemical processes in which the fluorophores emit photons from the excited states [10, 11]. Finally, the image-forming system relays a nearly exact image of the cell model to a light-sensitive detector.

#### Simulation of cell model

In particular, the bioimaging simulation system requires the space-time trajectory of each simulated molecule of interest to generate realistic digital images. However, many cell simulation systems have been designed to model and simulate both deterministic and stochastic biochemical processes, assuming that simulated molecules are dimensionless and homogeneously distributed in a compartment [12]. Here, we use spatial simulation methods that can provide accurate space-time trajectories of molecules [9, 13–17]. For a given cell system, simulations using these methods include a statistical model of biological fluctuation that arises from stochastic changes in the cellular compartment geometry, number of molecules, type of molecule, molecular state, and translational and rotational diffusion.

#### Simulation of optical system

Simulations of an optical system particularly require the computation of the photon counting, propagation, and distribution. The optics simulations are based on geometric optics (or wave optics) and the Monte Carlo method. Each optics simulation includes a statistical model of the systematic effects that are influenced by the parameters defined in optical devices such as the light source, objective lens, special filter, and detector. The classical theory of geometric optics is applied to simulate the photon propagation and distribution through the illumination and image-forming systems, including optical aberrations. Geometric optics approximates the photon propagation as a ray (paraxial approximation), and provides the procedures to compute the numerical or analytical forms of the photon distributions for a given photon wavelength. It is an excellent approximation when the photon wavelength is very small compared with the size of the structure with which the photon interacts. However, it introduces normalization constant as an input parameter, and is formalized without counting the number of photons propagating through the optical system. The Monte Carlo method is applied to the simulation of the stochastic process of counting photons for a given probability density function. The details for each optics simulation are described below.

1. Illumination system [18, 19]: The bioimaging system requires intense, near-monochromatic, illumination by a widely spreading light source, such as lasers. Incident photons from such a light source can illuminate a specimen. The surviving photons after passing the excitation filters interact with the fluorophores in the cell model, and excite the fluorophores to electrically excited states. The optics simulations of the focusing of the incident photons through the objective lens include a statistical model of the systematic effects due to the numerical aperture (NA), magnification, working distance, degree of aberration, correction refracting surface radius, thickness, refractive index, and details of each lens element.
2. Molecular fluorescence: The incident photons propagating through the illumination system are absorbed by the fluorophores in the cell model. Fluorescence is the result of physical and chemical processes in which the fluorophores emit photons from electronically excited states [10, 11]. The Monte Carlo simulation of the overall fluorescence process includes a statistical model of the systematic effects that are influenced by the absorption and emission spectra, quantum yield, lifetime, quenching, photobleaching and blinking, anisotropy, energy transfer, solvent effect, diffusion, complex formation, and a host of environmental variables.
3. Image-forming system [18, 19]: In an optical system that employs incoherent illumination of the cell model, the image-forming process can be considered as a linear system [20]. Impulse response of the image-forming system to a point-like fluorophore is described by the point spread function (PSF) of the wavelength and position. When all fluorophores in the cell model are imaged simultaneously, the distribution of emitted photons of longer wavelengths that passed through the use of the objective lens and special filters, is computed as the sum of the PSFs of all fluorophores. The optics simulations of PSF formation and convolution include a statistical model of the systematic effects that are ruled by the parameters embedded in the objective lens, the special filters, and each details of lens elements.
  The emitted photons are finally detected by light-sensitive devices, and digitized as an image at detection time. The properties of the final image depend on the detector specifications and conditions during the readout process that converts an incident photon signal into a digital signal. The Monte Carlo simulation for the detection process includes a statistical model of the systematic effects that arise from signal and background shot noises, and detector specifications and conditions, such as pixel size, quantum efficiency (QE), readout noise, dark current, excess noise factor, gain, offset, exposure time, and binning.

### Implementation

**Fig 2:**
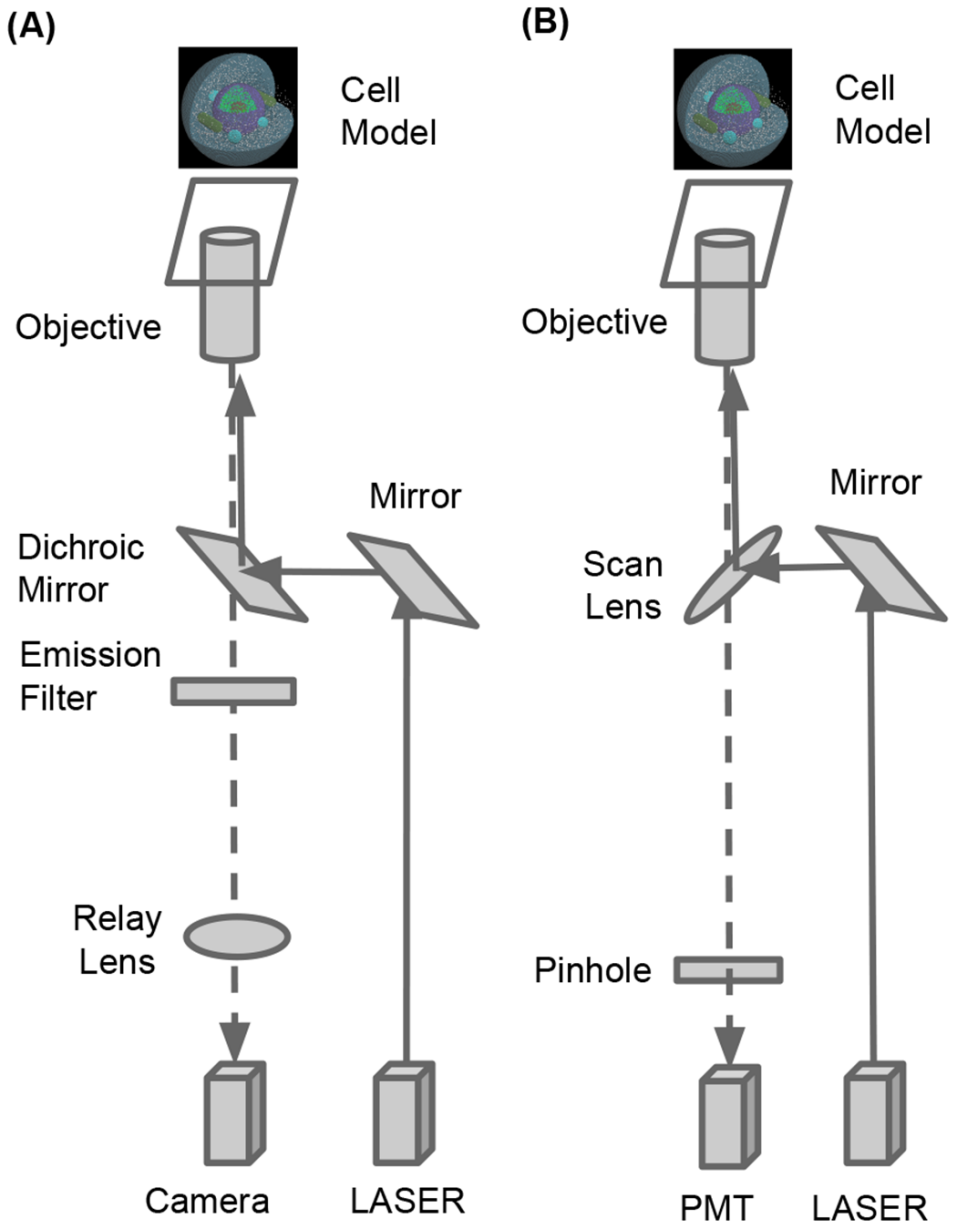
Optical configurations. (A) TIRFM simulation module. (B) LSCM simulation modules. Grey arrows represent direction of photon propagation.

We provide a standard computational framework to simulate various different types of bioimaing systems. In particular, we implemented the simulation modules for relatively simple microscopy systems: TIRFM and LSCM. Optical configurations are shown in Fig 2. The modules are designed to generate digital images of the cell simulation results after accounting for the systematic effects that are governed by the parameters defined in the TIRFM and LSCM systems. A cell simulation method with Spatiocyte is used to construct the spatial cell models [9]. For a given cell system, Spatiocyte can provide a statistical model of biological fluctuation that arises from stochastic changes in the cellular compartment geometry, number of molecules, type of molecule, molecular state, and translational diffusion. The method can be used to model complex reaction-diffusion mediated cellular processes occurring on the surface and in the volume compartments of the cell at a single-molecule resolution. To represent cell compartments and rapidly resolve molecular collisions, the method discretizes space into a hexagonal closed-packed lattice. Each molecule randomly walks from voxel to voxel. Molecular collisions occur between walks. Immobile lipid molecules represent surface compartments, such as cellular and nuclear membranes. Implementation details are described in ref. [21]. In addition, other simulation methods such as Green Function Reaction Dynamics (GFRD) [13, 14] will be applied in the future implementation.

The three dimensional point spreading function (3D-PSF) model plays a key role in the bioimaing simulations [22]. Each point-like source of a fluorophore gives rise to a 3D-PSF pattern in the image-forming systems. The normalization constant of the PSF is usually considered as an user input parameter. However, the bioimaging simulations requires the counting of the number of photons emitted from a single fluorophore, and spatial PSF integration to be unity within infinite volume region ($\int_{0}^{\infty }PSFd^{3}r=1$). The PSF decays in an oscillatory manner at tails along the radial and axial axes. Such damping characteristics hinders the estimation of an exact or approximate form of the PSF normalization constant. A wrong estimation can easily lead to the miscounting of the number of photons, and provide a wrong intensity of the final images. Such problematic normalization has not been well discussed in the literature. In addition, optical aberrations can lead to a non-uniform distribution of the 3D-PSF. The aberrations are deviations in an image that occur when photons from one point of an object does not converge into a single point after propagating through an optical system. They can be caused by artifacts that arise from the interaction of photons with glass lenses. Using first order paraxial approximation, makers of optical instruments typically correct the optical systems to compensate for the optical aberrations.

Assuming the first order paraxial approximation, and the spatial PSF integration to be unity within a limited volume region ($\int_{0}^{\Lambda }PSFd^{3}r=1$), we implement the TIRFM and LSCM simulation modules. Step-by-step instructions are provided below. More details are discussed in the supporting information (see S1 Text). Simulation studies to estimate the errors that arise from the PSF normalization and the optical aberrations are required for the future implementation.

A1 The TIRFM simulation module enables selective visualization of the basal surface regions of the cell model. Incident beam photons of the excitation wavelength (*λ*) can uniformly illuminate the specimen. Evanescent field is generated along z-axes as perpendicular to the total internal reflection surface, and capable of exciting the fluorophores near the surface. The incident photon flux density at the level of photon-counting unit is defined by
  $$\begin{array}{c} {\left|A_{I}\right|}^{2}≅\frac{\phi }{E_{\lambda }}\;[\frac{\#\text{photons}}{\;\text{sec}\cdot {\text{cm}}^{2}}] \end{array}$$ (1)
  where *ϕ* and $E_{\lambda }=\frac{hc}{\lambda }$ are the incident beam flux density (W/cm^2^) and single photon energy, respectively. *h* and *c* are Planck constant and a speed of light. **A**_*I*_ is the amplitude of the incident photon flux density.
A2 Because of the desperate timescales of the quantum transitions, we simply assume that the fluorescence molecules subsequently emit single photon of longer wavelength while they absorb one million photons of excitation wavelength, and the cross-section of photon-molecule interaction is roughly 10^−14^ cm^2^ [23]. No other physical processes is simulated. The expected number of photons emitted from a single fluorophore is defined by
  $$\begin{array}{ccc} n_{emit} & ≅ & \frac{\sigma \;\delta T}{4\pi }\;{\left|A_{T}\right|}^{2}\times 10^{-6}\;[\#\text{photons}] \end{array}$$ (2)
  where ∣**A**_*T*_∣^2^, *σ*, and *δT* are the transmitted beam flux density, the cross-section, and detection time. The detector is located in a specific direction. We expect to observe the number of photons devided by an unit surface area of a sphere (4*π*). The amplitude of the transmitted beam flux density depends on the index refraction, and the incident beam angle, amplitude, and polarization.
A3 When all the fluorophores in the cell model are imaged simultaneously, the distribution of the emitted photon of longer wavelengths that passed through the use of objective lens and special filters is computed as the sum of the PSFs of all the fluorophores. In particular, we use the Born-Wolf PSF model [22]. For an optimal wavelength (*λ*′) of a fluorophore, we estimated that 55% of the emitted photons that passed through the Dichroic mirror and emission filter survive ($n_{emit}\to n_{emit}^{\prime }$). The expected image plane at the focal point (*z* = *z*_0_) is given by the convolution of the PSF and written in the form of
  $$\begin{array}{c} Exp.\;Image\left(\vec{r},z\right)=\sum_{k=0}^{N}n_{emit}^{\prime }\;PSF_{\lambda^{\prime }}\left(\vec{r}-\frac{{\vec{r}}_{k}}{M},z-z_{k}\right) \end{array}$$ (3)
  where *N* and *M* are the total number of fluorophores, and optical magnification, respectively. $\left({\vec{r}}_{k},z_{k}\right)$ is the position of the *k*-th fluorophore. $\left(\vec{r},z\right)$ is the position in an image plane. The PSF is normalized within a ±1.0 *μ*m range of radial and axial axes. In addition, polarization of the evanescent field is non-isotropic, which means that dipoles of different orientations are excited with different probabilities per unit time. In order to accurately simulate image-formation process, the polarized form of the PSF is required for the future implementations.
A4 The emitted photons are finally detected by CMOS or EMCCD cameras, and digitized as an image at a detection time. The readout process can convert expected incident photon signals to digital signals relies on camera specifications and camera operating conditions to carry out the properties for final images. The observed image of the cell model can be obtained using the Monte Carlo method in the presence of systematic sources, including statistical fluctuations in photon counting (photon shot noise), and camera specification and camera operating conditions. Finally, photoelectron signals can be linearly converted to digital signals. Unit conversions are given by
  $$\begin{array}{c} Exp.\;Image\;[\#\text{photons}]⟶Obs.\;Image\;[\#\text{photoelectrons}]⟶Digital\;Image\;[A/D\;\text{counts}] \end{array}$$

B1 The LSCM simulation module can visualize focal regions of the cell model. In general, laser beam propagation of excitation wavelength can be approximated by assuming that the laser beam has an ideal Gaussian beam profile. The incident beam flux of excitation wavelength (*λ*) and continuously illuminates specimen, and is focused into a confocal volume at a given scan time and beam position. Incident photon flux is defined by
  $$\begin{array}{c} P^{\prime }≅\frac{\Phi }{E_{\lambda }}\;[\frac{\#\text{photons}}{\text{sec}}] \end{array}$$ (4)
  where Φ and $E_{\lambda }=\frac{hc}{\lambda }$ are the incident beam flux (W) and single photon energy. *h* and *c* are Planck constant and speed of light, respectively.
B2 We also assume that the linear conversion of photon emission is by 10^−6^, and the cross-section of photon-molecule interaction is roughly 10^−14^ cm^2^ [23]. No other physical processes are simulated. For a given position and time, the expected number of photons emitted from a single fluorophore is defined by
  $$\begin{array}{ccc} n_{emit}\left(\vec{r},z\right) & ≅ & \frac{\sigma \;\delta T}{4\pi }\;I\left(\vec{r},z\right)\times 10^{-6}\;[\#\text{photons}] \end{array}$$ (5)
  where *I*(*r*,*z*), *σ*, and *δT* are the transmitted beam flux density, cross-section, and scan time per pixel, respectively. The detector is located in a specific direction. We expect to observe the number of photons divided by an unit surface area of a sphere (4*π*). The transmitted beam flux density depends on the incident photon flux, and the beam waist radius at the focal plane where the wavefront is assumed to be flat.
B3 When all the fluorophores in the cell model are imaged simultaneously, the distribution of the emitted photon of longer wavelengths that passed through the use of objective lens and pinhole is computed as the sum of the PSFs of all the fluorophore. In particular, we use the Born-Wolf PSF model [22]. As an incident beam is scanned across the cell model in horizontal and vertical axes, a digital image is generated at a time. For a given scan time and beam central position, the expected image plane at the focal point (*z* = *z*_0_) is given by the integration of the image plane obtained from the PSF convolution. It is written in the form of
  $$\begin{array}{ccc} Exp.\;Image(\vec{r},z) & = & \;\int \int \;\delta \left({\vec{r}}_{b}-\vec{r},z_{b}-z\right)[{\;\int \int \;}_{|\vec{r^{\prime }}-{\vec{r}}_{b}|<R}I^{\prime }\left(\vec{r^{\prime }}-{\vec{r}}_{b},z^{\prime }-z_{b}\right)\;dx^{\prime }dy^{\prime }]dx_{b}dy_{b} \end{array}$$ (6)
  $$\begin{array}{c} \text{where}\;I^{\prime }\left(\vec{r^{\prime \prime }},z^{\prime \prime }\right)≅\sum_{k=0}^{N}n_{emit}\left(\vec{r^{\prime \prime }},z^{\prime \prime }\right)\;PSF_{\lambda^{\prime }}\left(\vec{r^{\prime \prime }}-\frac{{\vec{r}}_{k}}{M},z^{\prime \prime }-z_{k}\right) \end{array}$$
  where *N*, *R* and *M* are the total number of fluorophores, pinhole radius, and optical magnification, respectively. $\left({\vec{r}}_{k},z_{k}\right)$ is the position of the *k*-th fluorophore. $\left({\vec{r}}_{b},z_{b}\right)$ is the position of beam center. $\left(\vec{r},z\right)$ is position in the image plane. The PSF is normalized within a ±1.0 *μ*m range of radial and axial axes.
B4 The emitted photons are finally detected by a photomultipliers tube (PMT), and digitized as an image at a given scan time. The observed image of the cell model can be obtained using the Monte Carlo method in the presence of systematic sources, including statistical fluctuations in photon counting (photon shot noise), and PMT specifications and PMT operating conditions. Finally, photoelectron signals can be linearly converted to digital signals. Unit conversions are given by
  $$\begin{array}{c} Exp.\;Image\;[\#\text{photons}]⟶Obs.\;Image\;[\#\text{photoelectrons}]⟶Digital\;Image\;[A/D\;\;\text{counts}] \end{array}$$

## Results

### Comparison of *in vitro* images

**Fig 3:**
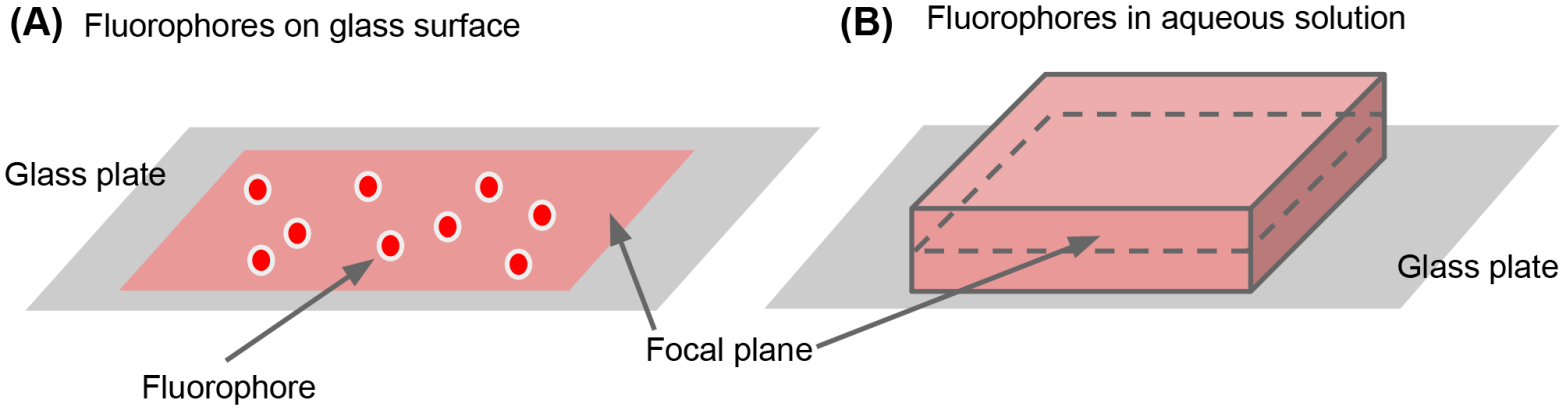
Simple models (A) 100 stationary HaloTag-TMR molecules are distributed on a glass surface. (B) 19,656 HaloTag-TMR molecules are distributed in a 30 × 30 × 6 *μ*m^3^ box of aqueous solution (= 5 nM), and rapidly diffuse at 100 *μ*m^2^/sec.

**Fig 4:**
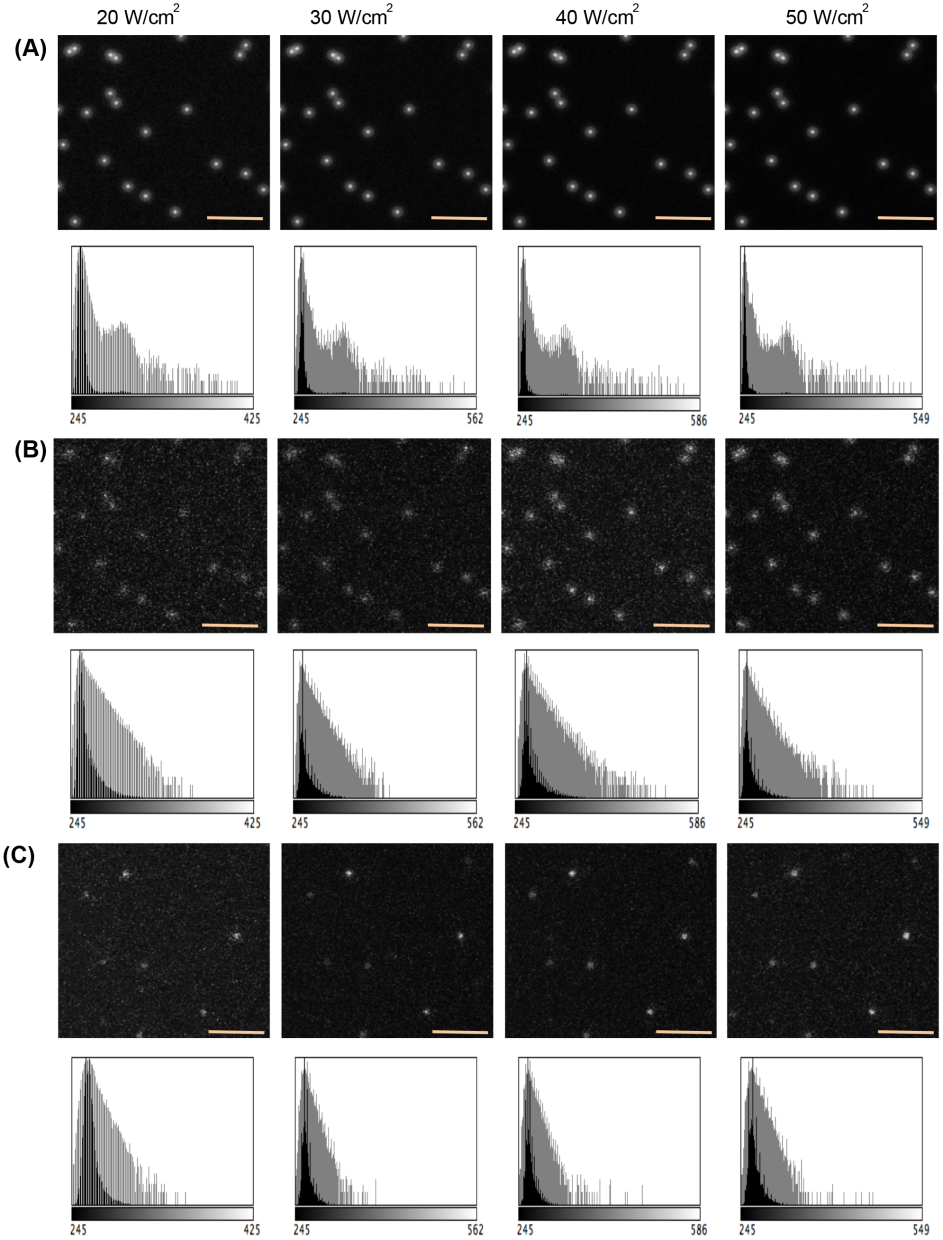
Using HaloTag-TMR molecules distributed on a glass surface to evaluate the performance of TIRFM simulation module. (A) Expected images of the simple particle model at various beam flux densities (20,30,40 and 50 W/cm^2^). The expected images are obtained by averaging 100 images over 3 sec exposure period. Intensity histograms are also shown below each expected images and presented with black-colored bars. Each histograms are logarithmically scaled and presented with grey-colored bars. (B) Simulated digital images of the simple particle model are shown at various beam flux densities (20,30,40 and 50 W/cm^2^). Size of each images is 152 × 156 pixel. Orange scalebar represents 3.15 *μ*m. Intensity histograms are also shown below each simulated images. (C) Real captured images obtained from *in vitro* experiment are shown at various beam flux densities (20,30,40 and 50 W/cm^2^). The maximum value of the grayscale is adjusted to improve visualization of each image. Intensity histograms are also shown below each actual images.

**Fig 5:**
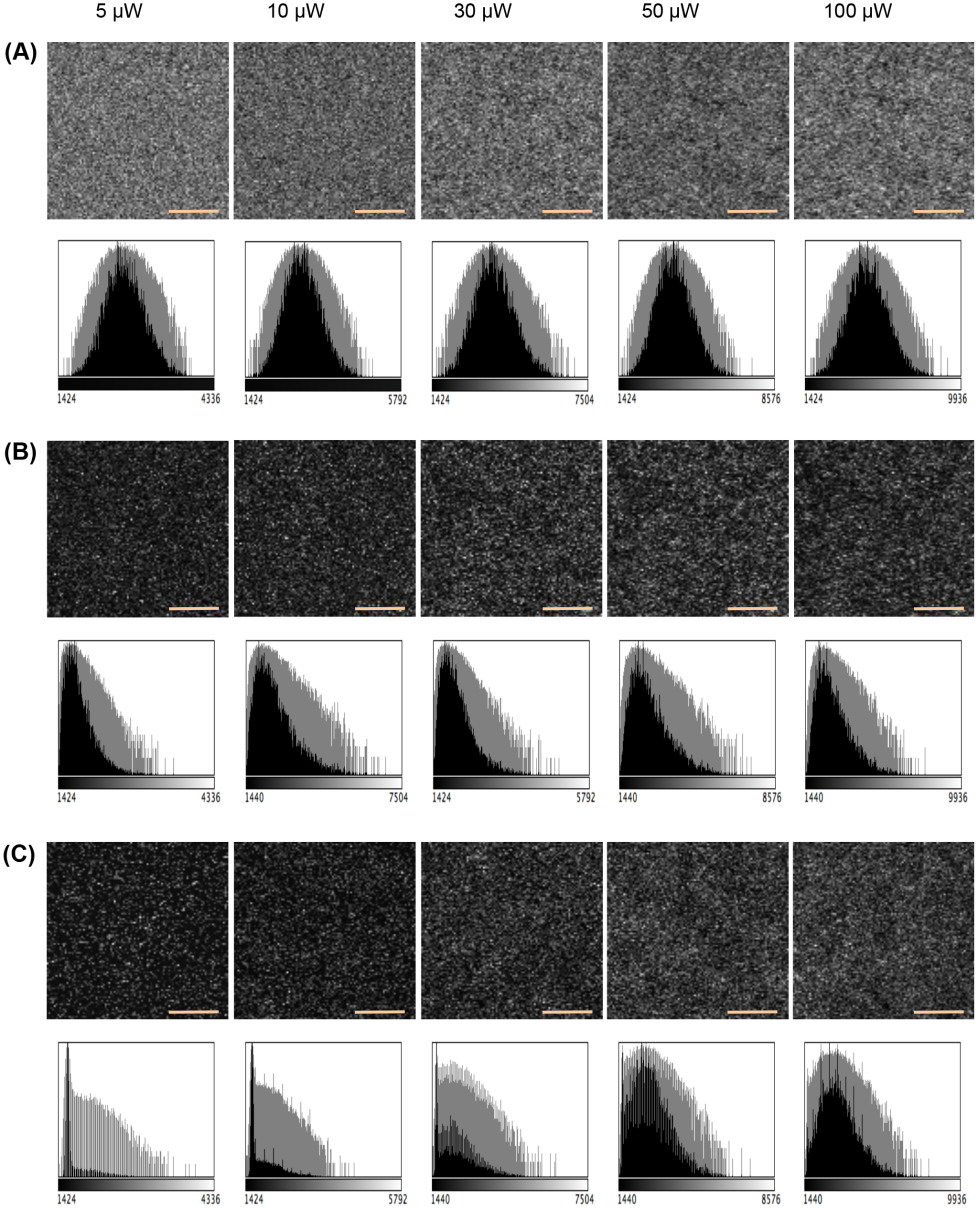
Using HaloTag-TMR molecules to evaluate the performance of LSCM simulation modules. (A) Expected images of the simple particle model at various beam flux (5,10,30,50, and 100 *μ*W). Each expected images are generated by averaging 30 images over 30 sec exposure period. Intensity histograms are also shown below each expected images and presented with black-colored bars. Each histograms are logarithmically scaled and presented with grey-colored bars. (B) Simulated digital images of the simple particle model are shown for various beam flux (5,10,30,50, and 100 *μ*W). Size of each images is 100 × 100 pixel. Orange scalebar represents 5.18 μm. Intensity histograms are also shown below each simulated images. (C) Real captured images obtained from *in vitro* experiment are shown for various beam flux (5,10,30,50, and 100 *μ*W). Size of each images is 100 × 100 pixel. The maximum value of the grayscale is adjusted to improve visualization of each image. Intensity histograms are also shown below each actual images.

We evaluated the performance of our simulation modules by comparing the simulated images with the actual photographed ones for simple particle models of fluorescent molecules. We simulated imaging of the focal region of those simple models for the optical system with the detector specifications and detector operating (see S2 Text). Details of the *in vitro* comparison are described in the supporting information (see S2 Text). The results are shown in Figs 3, 4 and 5. The intensity of the simulated images corresponds to the number of photons detected in the digital cameras or the PMT. Each simulated image is visually similar to the corresponding real ones. Thus, the simulated images were compared with images obtained using actual microscopy systems at the level of photon-counting units. However, differences still remain in the resulting images owing to calibration. Calibration is the process of improving the agreement of the code calculation with a chosen set of benchmarks through the adjustment of the parameters implemented in the simulation modules [6–8]. Such a calibration process is required in all experiments to improve the agreement of the simulated outputs with the *in vitro* data sets. Even though the results of a simple calibration were used, the first version of our simulation modules was capable of generating images that closely reproduce images obtained with an actual microscopy system. A more elaborate set of calibration is required in the future. More details are described below.

1. To test the performance of the TIRFM simulation module, we constructed a simple particle model of 100 stationary HaloTag-with-tetramethylrhodamine (HaloTag-TMR) molecules distributed on a glass surface, as shown in Fig 3A. We simulated imaging of the basal region of the simple model for the TIRFM specifications and TIRFM operating conditions (see S2 Text). Fig 4A shows the expected optical distribution used for the simulation, which was generated by averaging 100 images over a 3 sec exposure period. Intensity histograms of each expected images are also shown in Fig 4A. Fig 4B and 4C show the simulated images and the real captured ones at various beam flux densities. The intensity of the simulated images corresponded to the number of photons detected in the EMCCD camera. Increasing the beam flux density results in a relatively brighter image. Each simulated image is visually similar to the corresponding real one. Thus, the simulated images were compared with the images obtained using the actual TIRFM systems at the level of photon-counting units. However, differences still remain in the resulting images owning to calibration. A more elaborate set of calibrations is required in the future.
2. To test the performance of the LSCM simulation module, we constructed a simple particle model of 19,656 HaloTag-TMR molecules diffused in an aqueous solution as shown in Fig 3B. We simulated imaging of the middle region of the simple model for the LSCM specifications and LSCM operating conditions (see S2 Text). Fig 4A shows the expected optical distribution used for the simulation, which was obtained by averaging 30 images over a 30 sec exposure period. Intensity histograms of each expected images are also shown in Fig 4A. Fig 5B and 5C show the simulated images and the real captured ones at various beam fluxes. The intensity of the simulated images corresponds to the number of photon pulses detected in the PMT. Increasing the beam flux results in relatively brighter image. Each simulated image is visually similar to the corresponding real ones. Thus, the simulated images were compared with the images obtained using the actual LSCM systems at the level of photon-counting units. However, differences still remain in the resulting images owning to calibration. A more elaborate set of calibrations is required in the future.

### Comparison of *in vivo* images

**Fig 6:**
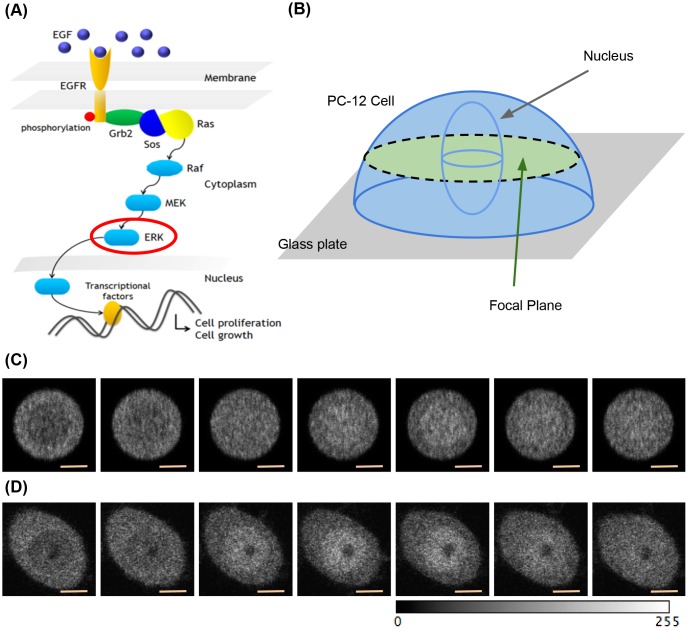
ERK nuclear translocation model of EGF signaling pathway. (A) Reaction network. (B) Geometry of PC-12 cell model. A hemispherical cell measuring 20 *μ*m in diameter and 7 *μ*m in height is assumed. (C) Time-lapse images of the ERK nuclear translocation model observed using the LSCM simulation module. Size of each images is 90 × 90 pixel. Orange scalebar represents 4.66 *μ*m. (D) The time-lapse images obtained from the experiment. The grayscale of each images is adjusted in the range of 0 to 225.

**Fig 7:**
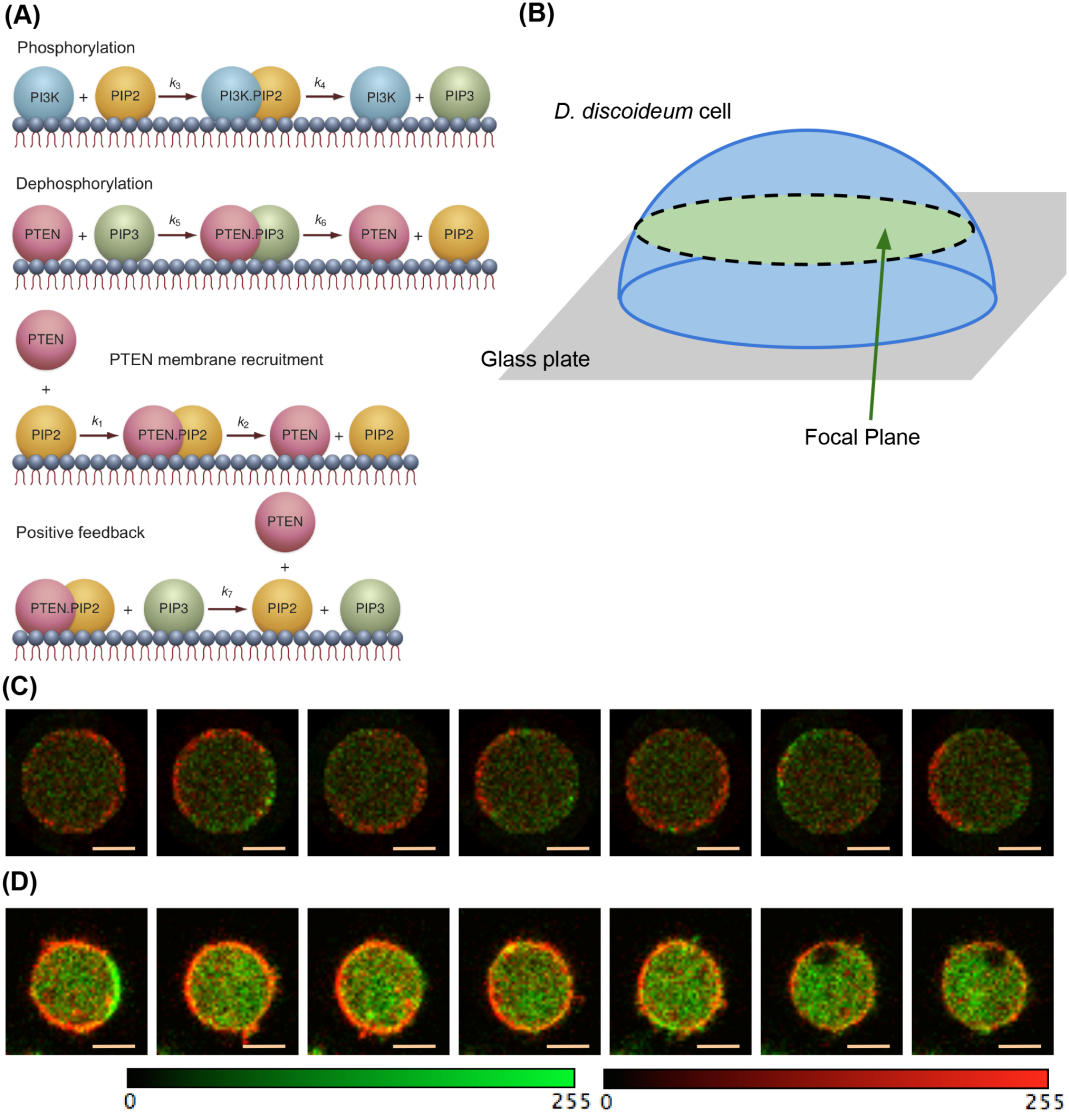
Self-organizing wave model of PTEN for the chemotactic pathway of *D. discoideum*. (A) Reaction network. (B) Geometry of *D. discoiduem* cell model. A hemispherical cell measuring 25 *μ*m in diameter and 5 *μ*m in height is assumed. (C) Time-lapse image of the self-organizing wave model observed using the LSCM simulation module. Size of each images is 52 × 51 pixel. Orange scalebar represents 5.39 *μ*m. (D) Time-lapse images obtained from the experiment. Red and green indicate PTEN-TMR and PH-EGFP, respectively. The colorscale of each images is adjusted in the range of 0 to 225.

Using the LSCM simulation module, we compared a more complex cell model with real cell images obtained using the actual LSCM system. We constructed the following spatial cell models: (i) the ERK nuclear translocation model for the EGF signaling pathway, and (ii) the self-organizing wave model of PTEN for the chemotactic pathway of *D. discoideum*. We developed these cell models, which are not available in the literature. We assumed that the parameters of each cell model and the LSCM system are well evaluated with *in vitro* data sets. We then simulated imaging of the focal region of those cell models for the LSCM specifications and LSCM operating conditions (see S3 Text). Details of the *in vivo* comparison are described in the supporting information (see S3 Text). The results are shown in Figs 6 and 7. The intensity of the simulated images corresponds to the number of photon pulses detected in the PMT. Thus, the simulated cell images were compared with the images obtained by the actual microscopy systems at the level of photon-counting units. Significant new insight on the cell models will be published in the future.

i. We constructed the cell model of ERK nuclear translocation for the EGF signaling pathway. We assumed the PC12 cell model that represents the ERK molecules tagged with the enhanced green fluorescent protein (ERK-mEGFP). Fig 6A and 6B show the main reaction network and the geometry of the model, respectively. The cell was placed on the glass surface, and was nearly hemispherical. The size of the hemispherical cell was estimated by experimentalists. A cell measuring 20 *μ*m in diameter and 7 *μ*m in height was assumed. The model consisted of 75 chemical species, 143 reactions, and 85 kinetic parameters. A maximum of 100,000 ERK molecules were distributed in the cell cytoplasm and rapidly diffuse at 1.00 *μ*m^2^/sec. The input of the EGF ligand could drive the transport of 30% of the ERK molecules into the nucleus and back to the initial condition in 10 min. We simulated imaging of the middle regions of the cell model for the LSCM specifications and LSCM operating conditions (see S3 Text). Fig 6C and 6D show the simulated cell images and the cell images obtained using the actual LSCM system. The intensity of the simulated images corresponds to the number of photon pulses detected in the PMT. Therefore, the simulated images were compared with images obtained using the actual LSCM system at the level of photon-counting units (see S1 Video). Each simulated image was visually similar to the corresponding real one, but differences still remain in the resulting images owning to calibrations. A more elaborate set of calibration is required in the future.
ii. We also constructed a self-organizing wave model of PTEN for the chemotactic pathway of *D. discoideum* to validate the performance of two-color imaging for the LSCM simulation module. We assumed a *D. discoideum* cell model that expresses the fluorescently labeled pleckstrin homology domain of Akt/PKB (PH) and PTEN, where PH and PTEN are indicators for phosphorylates phosphatidylinositol 3,4,5-trisphosphate (PIP3) metabolism. PH can bind to PIP3 at the membrane, whereas PTEN catalyzes the degradation of PIP3 and has a binding motif for phosphatidylinositol 4,5-biphosphate (PIP2). PH was tagged with EGFP (PH-EGFP), whereas PTEN was tagged with HaloTag with TMR (PTEN-TMR). A maximum of 10,000 molecules of PTEN-TMR and PH-EGFP were homogeneously distributed in the cell cytoplasm. On the membrane, PI3K catalyzed PIP2 phosphorylation to PIP3, whereas PTEN dephosphorylated PIP3 into PIP2. Cytosolic PTEN was recruited to the membrane regions containing PIP2. Nonetheless, PIP3 could dislodge PTEN from PIP2 into the cytosol when they came in contact with each other. This last reaction acted as a positive feedback for PIP3 accumulation. Fig 7A and 7B show the main reaction network and the geometry of the model, respectively. A cell was placed on the glass surface, and was nearly hemispherical. The size of the hemispherical cell was estimated by experimentalists. The cell measuring 25 *μ*m in diameter and 5 *μ*m in height was assumed. The model involved 8 chemical species, 12 reactions, and 12 kinetic parameters. Lattice-based particle simulation of the cell model enabled of the reproduction of the local oscillatory dynamics of PTEN-TMR and PH-EGFP. We simulated imaging of the middle region of the cell model for the LSCM specifications and LSCM operating conditions (see S3 Text). Fig 7C and 7D show the simulated cell images and the cell images obtained by the actual LSCM system. The intensity of the simulated images corresponds to the number of photon pulses detected in the PMT. Therefore, the simulated images were compared with the images obtained using the actual LSCM system at the level of photon-counting units (see S2 Video). Each simulated image was visually similar to the corresponding real one, but intensity differences still remained in the resulting images. The number of PTEN-TMR and PH-EGFP in the wave model are approximately 4,000 for each, but we expect more (∼30,000) in the observed images. A more elaborate set of calibration is required in the future.

## Discussion

Measurements using bioimaging techniques are generally influenced by systematic effects that arise from the stochastic nature of biological cells, the photon-molecule interaction, and the optical configuration. Such systematic effects are always present in all bioimaging systems and hinder the comparison between the cell model and the real cell image. Combining optics and cell simulation technologies, we proposed a computational framework for handling the parameters embedded in the cell model and the optical principles governing the bioimaging systems. The simulation using this framework generated digital images from cell simulation results after accounting for the systematic effects. In particular, we demonstrated that the simulated digital images are visually similar to the images obtained using actual TIRFM and LSCM systems. Each pixel intensity corresponded to the number of photon pulses detected in the camera or the PMT. Thus, the framework streamlines the comparison at the level of photon-counting units. However, the image comparison is insufficient to check the validity of the simulation modules. Verification is the process of confirming the simulation modules are correctly implemented with respect to conceptual description and analytical solutions [6–8]. During the verification process, the simulation modules must be tested to find and estimate numerical errors in the implementations. The simulation modules are designed to count the number of photons that passed through the optical configurations. A wrong estimation of the numerical errors that arise from the photon-counting principle can provide a wrong intensity of the final images. For example, a wrong PSF normalization can miscount the number of photons, and lead to wrong final images. Furthermore, the simulated images can be also compared with a chosen set of experimental benchmarks defined in calibration and validation parameter domains [6–8]. Systematic variance and covariance that arise from various different parameter settings must be estimated to establish the validity of the simulation modules. Analyses to quantify the systematic uncertainties are required for the future implementation.

One of the key challenges of transforming biology from a phenomenological science to a predictive one is how to bridge the gap between a cell model and an actual biological cell [24–28]. Over the last two decades, large-scale, accurate, and comprehensive simulations of cell models have greatly improved our understanding of many cellular networks and processes [29–31]. However, we are still far away from having predictive cell models for actual applications in medicine and biotechnology. In this work, we focused on the “comparison” part of the model validation and demonstrated the single cell-to-cell image comparison at the level of photon-counting units. For future implementation, it is important to fully simulate optical systems and to demonstrate other important parts of the model validation [6–8]. Within this framework, the functionality and capability of the cell models will be more easily seen and understood. Future tasks required for the model validation include studying diversity in cell populations and obtaining the nominal and predicted probability distributions of the cell model. The behavior of individual cells depends on the internal variables and the environmental conditions. The nominal and predicted probability distributions of those variables are characterized by their statistical quantities. A likelihood that quantifies the discrepancy between the predicted distribution and the observed one can be evaluated by using a statistical test of significance. If the result of the statistical test satisfies a certain confidence level, then the cell model is either rejected or accepted with respect to real cell images. Consequently, such model fitting will support discoveries in biological science.

Bioimaging simulation using the computational framework presented here is not meant to replace biological experiments. It provides a realistic estimate of the output that would be obtained in specific biological applications. Biologists often use commercial bioimaging systems for their own biological interests. Optical properties of biological molecules and/or phenomena uniquely change, according to the experimenter’s skills and experiences in handling biological samples and optical equipments. The commercial systems are designed for general usage, and are not optimized to measure the optical properties of all biological samples. Although some biologists assemble specialized optical imaging systems for a particular application, it is still difficult for them to adjust systems parameters without expected outputs. Such an approach is quite inefficient since it depends on the experimenter’s skills and experiences. A more systematic approach is required to reduce or eliminate unintended experimenter’s bias. In order to objectively handle biological and physical principles in an organized manner, it is important to develop an object-oriented simulation toolkit of biological imaging. The simulation toolkit is constructed on the basis of a set of numerous biological and physical processes to handle diverse interactions of photons with molecules over a wide energy range. The toolkit provides a complete set of software components for all area of bioimaging simulations: optical configuration, spatial cell models, run, parameter management, visualization and user interface. Such a multi-disciplinary nature of the toolkit allows a user to easily design, customize and extend bioimaging and/or experimental systems well optimized for specific biological applications. For example, the computational framework can also be applied to simulate other bioimaging techniques including fluorescence recovery after photobleaching (FRAP), fluorescence correlation spectroscopy (FCS), Forster resonance energy transfer (FRET) and localization microscopy. All simulation modules can be objectively handled in a uniform software platform.

However, there are two problems in constructing such a software platform. (1) Computational speed is not well optimized for the TIRFM and LSCM simulation modules. The speed of generating a simulated image is proportional to the number of fluorophores embbeded in a cell models. Bioimaging simulation of a cell model containing 100,000 fluorophores, requires about one day to obtain the final image. Optimization is required in the near future. (2) The optical properties of many commercial materials are not publicly available. In particular, information on the objective lens used is important for predicting an exact PSFs in a wide field. A question is how we can overcome such nonscience-related problems (probably, it is a matter of business model). In conventional approaches to biological research, biologists and optical physicists work independently, and do not interact much technologically. In order to properly design and customize the bioimaging and experimental systems well optimized for the specific biological applications, collaborative work with optical physicists and engineers will be required for the future biological research. Clearly, the bioimaging simulation toolkit allows us to better communicate with optical physicists and engineers, and to perform the simulation studies of bioimaging systems and their operating conditions. Optical materials are well designed by optical physicists and engineers, and their performance is generally validated by simulation studies of physical principles and their boundary conditions. Simulation studies are essential for the objective examination of the response of the optical equipments. However, such simulation studies have not been well performed for biological samples. Without the results of simulation studies for biological samples, the collaboration could easily fail. Then, information on the optical materials could not be shared. Using whatever form of PSF as realistically as possible, it is important to estimate experimental accuracy and precision for valuable discussion. We believe that the simulation toolkit can bridge the gap between biology and optics.

## Supporting Information

S1 Text Implementation details. (PDF)

S2 Text Details of *in vitro* image comparison. (PDF)

S3 Text Details of *in vivo* image comparison. (PDF)

S4 Text Parameterization for ERK nuclear translocation model. (PDF)

S5 Text Parameterization for self-organizing wave model of PTEN. (PDF)

S1 Video *In vivo* image comparison movie for ERK nuclear translocation model. (MOV)

S2 Video *In vivo* image comparison movie for self-organizing wave model of PTEN. (MOV)
