# Supplementary material for: A Computational Framework for Bioimaging Simulation
Source: PLoS One. 2015 Jul 6;10(7):e0130089. doi: 10.1371/journal.pone.0130089 (PMC4509736; doi:10.1371/journal.pone.0130089)
Supplement: S1 Text — (PDF) [file pone.0130089.s001.pdf]

Text S1: Implementation details

Abstract

This supporting information provides implementation details of the TIRFM and LSCM simulation modules. Implementation is generally not practical and requires much time. For the first implementation, we often applied simple theoretical formulas to simulate the illumination system, molecular fluorescence and PSF formation. We are planning to fully simulate the optical systems for future implementation. The complete source code of these simulation modules was written in Python and released as an open-source framework at `https://github.com/ecell/bioimaging`. The package is freely available for Linux and Mac OS X.

Contents

1 Implementation **2**
- 1.1 TIRFM simulation module 3
  - 1.1.1 Illumination system 3
  - 1.1.2 Molecular fluorescence 5
  - 1.1.3 Image-forming system 6
  - 1.1.4 Examples of images 10
- 1.2 LSCM simulation module 20
  - 1.2.1 Illumination system 20
  - 1.2.2 Image-forming system 21
  - 1.2.3 Examples of outputs 23

1 Implementation

We proposed a standard computational framework to simulate the passage of photons through fluorescent molecules and the optical system, and to generate a digital image that closely represents the image obtained using an actual fluorescence microscopy system. The computational framework included a statistical model of the systematic effects that are influenced by the parameters defined in the cell model and optical system. Using this framework, we specifically implemented the simulation modules for relatively simple bioimaging systems: TIRFM and LSCM techniques. Optical configurations are shown in Figures S1.1 and S1.22. Those modules were designed to generate digital images that closely represent the actual digital images obtained using actual TIRFM and LSCM systems. The optics simulation of the passage of photon through the microscopy systems was based on either geometric optics or the Monte Carlo method. The optics simulation is composed of three components; (1) illumination system, (2) molecular fluorescence and (3) the image-forming system. The illumination system transfers the photon flux from a light source to a cell model, to generate a prescribed photon distribution and maximize the flux delivered to the cell model. Fluorophores defined in the cell model absorb photons from the distribution, and are quantum-mechanically excited to higher energy states. Molecular fluorescence is the result of physical and chemical processes in which the fluorophores emit photons in the excited state. Finally, the image-forming system relays a nearly exact image of the cell model to the light-sensitive detector.

Implementations of the simulation modules are generally not practical and require much time. Assuming the first-order paraxial approximation and the spatial PSF integration to be unity within a limited volume region ($\int_0^\Lambda PSF\ d^3r = 1$), we implement the TIRFM and LSCM simulation modules. Theoretical formulas are often applied in the first implementation. Simulation studies to estimate the errors that arise from the PSF normalization and optical aberrations are required for future implementation.

1.1 TIRFM simulation module

The TIRFM simulation module enables a selective visualization of basal surface regions of a cell model. Its optical configuration is shown in Figure S1.1 [35, 33]. Implementation assumption are summarized in Table S1.1.

Principle	Photon-counting<br>1st-order paraxial approximation (Linear term)
Illumination	Epi-fluorescent or Evanescent fields<br>Continuous / Uniform / Linearly-polarized
Fluorescence	Linear convertion ($\times 10^{-6}$)<br>Cross-section ($\sigma \cong 10^{-14}$ cm^2)
Image-forming	3-D Airy PSF (Unpolarized analytical form)<br>CMOS or EMCCD cameras

S1 Table S1.1: Implementation assumptions for the TIRFM simulation module. The detection process for the cameras is performed with Monte Carlo simulation, where CMOS and EMCCD stand for complementary metal-oxide semiconductor and electron multiplication charge coupled device, respectively.

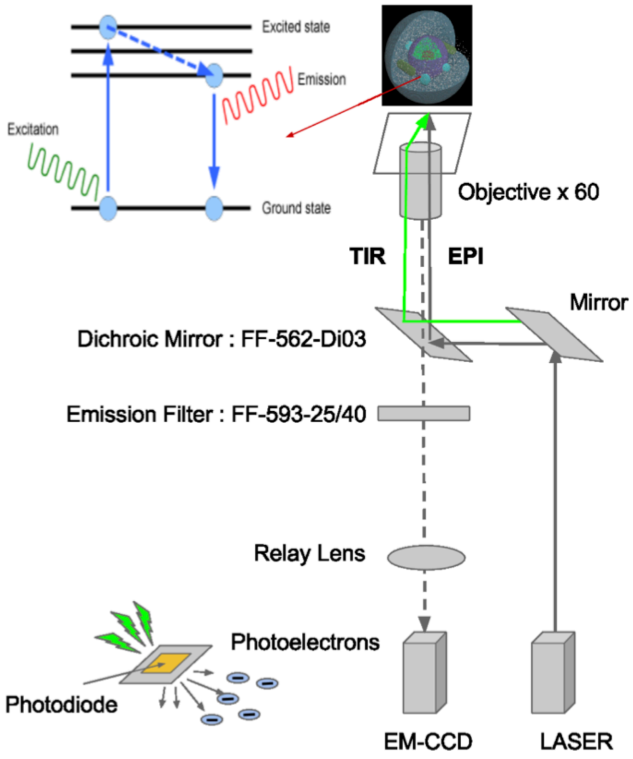

S1 Fig. S1.1: Optical configurations of the TIRFM simulation module

1.1.1 Illumination system

An incident beam of excitation wavelength (λ) that passed through the objective lens is assumed to uniformly illuminate specimen. The survived photons through the use of excitation filters interact with the fluorophores in the cell model, and excite the fluorophores to the electrically excited state. The optics simulations for the focusing of the incident photons through the objective lens include a

statistical model of systematic parameter rules by specifications including numerical aperture (NA), magnification, working distance, degree of aberration, correction of refracting surface radius, thickness, refractive index and details of each lens element. Details of the illumination optics are described in refs. [16, 17].

The incidence angle of the beam is particularly important for the TIRFM system. Figure S1.2 illustrates schematic views of the evanescent field and epifluorescence fields with respect to different incidence beam angles. If the incidence angles are less than the critical angles given by $\sin\theta_c = n_2/n_1$, then most of the incidence beam propagates through the interface into the lower index material with a refraction angle given by Snell's Law. However, if the incidence angle is $\theta > \theta_c$, then the incidence beam undergoes total internal refraction (TIR). The evanescent field is generated along the z-axis as perpendicular to the TIR surface, and is capable of exciting the fluorescent molecules near the surface. The intensity of the evanescent field at any position exponentially decays with z, and is written in the form of

$$I(z) = |\mathbf{E}_T|^2 = |\mathbf{A}_T|^2 \exp\left(-\frac{z}{d}\right) \tag{1}$$

$$d = \frac{\lambda}{4\pi\sqrt{n_1^2 \sin^2\theta - n_2^2}} \tag{2}$$

where $\mathbf{E}_T$ and $\mathbf{A}_T$ are the transmitted electric field and amplitude of the incident beam as a function of incident beam angle, respectively. d and λ are the penetration depth of the evanescent field and the wavelength of the incident beam in vacuum, respectively.

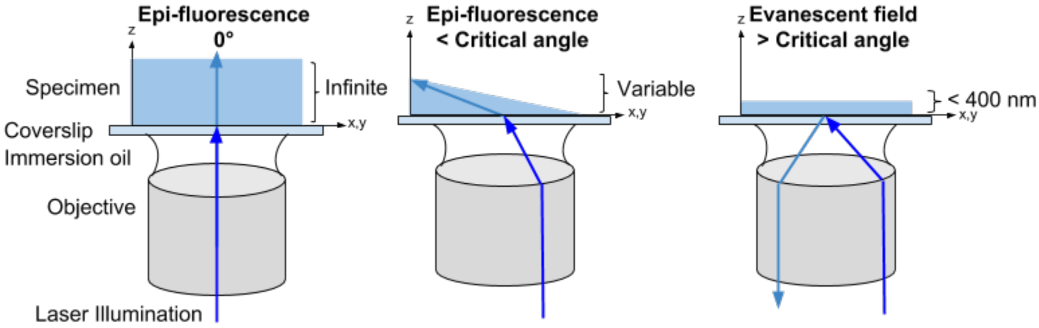

S1 Fig. S1.2: Epifluorescence (Left/Middle) and evanescent field (Right)

The polarization of the evanescent field depends on the incident beam polarization, which can be either p-pol (polarized in the plane of the incidence formed by the incident and reflected rays, denoted here as the x-z plane) or s-pol (polarized normal to the plane of incidence; here, in the y-direction). In both polarizations, the evanescent field fronts travel parallel to the surface in the x-direction. The p-pol evanescent field is a mixture of the transverse (z) and longitudinal (x) components; this distinguishes the p-pol evanescent field from freely propagating subcritical refracted light, which has no component longitudinal to the direction of travel. The longitudinal x component of the p-pol evanescent field range diminishes back toward the critical angle.

$$A_{Tx} = \frac{2\cos\theta\sqrt{\sin^2\theta - n^2}}{\sqrt{n^4\cos^2\theta + \sin^2\theta - n^2}} A_{Ip}\, e^{-i(\delta_p + \pi/2)} \tag{3}$$

$$A_{Tz} = \frac{2\cos\theta\sin\theta}{\sqrt{n^4\cos^2\theta + \sin^2\theta - n^2}} A_{Ip}\, e^{-i\delta_p} \tag{4}$$

For the s-pol evanescent field, the evanescent electric field vector direction remains purely normal to the plane of incidence (y-direction).

$$A_{Ty} \quad = \quad \frac{2\cos\theta}{\sqrt{1-n^2}}\, A_{Is}\, e^{-i\delta_s} \tag{5}$$

where AI is the field amplitude of the polarized incident beam. The phases relative to the incident beam are written as follows.

$$\delta_p \quad = \quad \tan^{-1}\left[\frac{\sqrt{\sin^2\theta - n^2}}{n^2\cos\theta}\right] \tag{6}$$

$$\delta_s \quad = \quad \tan^{-1}\left[\frac{\sqrt{\sin^2\theta - n^2}}{\cos\theta}\right] \tag{7}$$

The incident electric field amplitude in the substrate is normalized to unity for each polarization. More details are described in refs. [32, 33, 34, 35, 36].

1.1.2 Molecular fluorescence

Incident photons of specific wavelengths are absorbed by the fluorophores in the cell model. Fluorescence is the result of physical and chemical processes in which the fluorophores emit photons in the electronically excited state. The excitation of the fluorophores by an incident beam photon occurs in femtoseconds. Vibrational relaxation of excited-state electrons to the lowest energy state is much slower and can be measured in picoseconds. The fluorescence process (that is, emission of a longer-wavelength photon and the return of the molecule to the ground state) occurs in a relatively long time of nanoseconds. However, the process of phosphorescence from the triplet state and back to the ground state occurs in a much longer time of microseconds. A Jablonski diagram of the fluorescence process is shown in Figure S1.3. The Monte Carlo simulation of the overall fluorecence process includes a statistical model of systematic parameters ruled by the observable changes in absorption and emission spectra, quantum yield, lifetime, quenching, photobleaching and blinking, anisotropy, energy transfer, solvent effects, diffusion, complex formation, and a host of environmental variables. Details are described in refs.[18, 19].

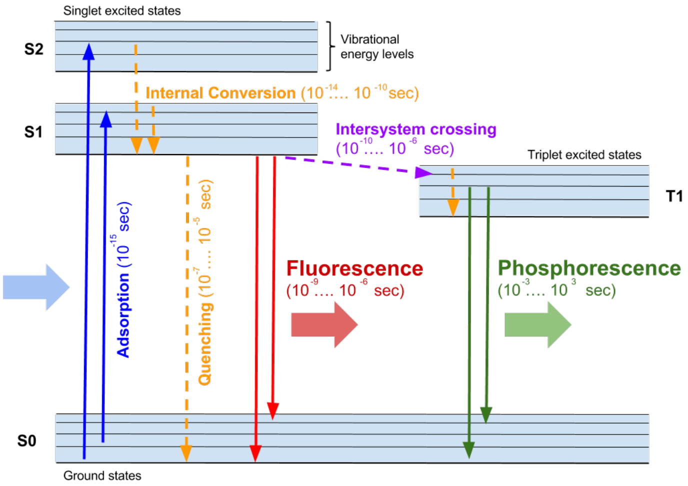

S1 Fig. S1.3: Jablonski diagram of molecular fluorescence and phosphorescence

1.1.3 Image-forming system

In an optical system that employs incoherent illumination of the cell model, the image-forming process can be considered as a linear system. The impulse response of an image-forming system to a pointlike fluorophore is described by the point spread function (PSF) of wavelength and position. The optics simulations of PSF formation and convolution include a statistical model of systematic parameters ruled by the observational changes in specifications of the objective lens and special filters. When all the fluorophores in the cell model are imaged simultaneously, the distribution of emitted photons of longer wavelengths passing through the objective lens and special filters used is computed as the sum of the PSFs of all the fluorophores. In the TIRFM system, the incident light that excites the fluorescent molecules is an evanescent field generated under the total internal reflection. The polarization of this light is non-isotropic, which means that dipoles of different orientations are excited with different probabilities per unit time. Therefore, the PSF of a fluorescent molecule should be written in the polarized form of the weighted average over orientations. Here, we use a simple analytical form of the unpolarized PSF models. The number of outgoing photons can spread depending on the PSF. The analytical forms of the PSF models are well studied in ref. [22] and is generally written as

$$\mathrm{PSF}_{\lambda'}(r,z) = \left| C \int_0^1 J_0(\alpha \rho r) \exp\left(-j\psi\right) \rho d\rho \right|^2 \tag{8}$$

where $\alpha = \frac{2\pi}{\lambda'} \frac{\mathrm{N.A.}}{n}$. $\lambda'$and n are fluorophore wavelength and the refractive index of the immersion layer. The phase factor, $\psi = \psi(r, z, \rho)$ enables generating the second Airy peak along the z-axis. In particular, we use Born-Wolf PSF model given by

$$\mathrm{PSF}_{\lambda'}(r,z) = \left| C \int_0^1 J_0(\alpha \rho r) \exp\left(-j\beta\rho^2 z\right) \rho d\rho \right|^2 \tag{9}$$

where $\alpha = \frac{2\pi}{\lambda'} \frac{\mathrm{N.A.}}{n}$, $\beta = \frac{1}{2}\frac{2\pi}{\lambda}\left(\frac{\mathrm{N.A.}}{n}\right)^2$ and C is the normalisation factor. In this PSF model, when the particle is in focus, the scalar-based diffraction can occur in the microscopy system, but the imaging plane is not required to be in focus. The model is shift invariant in all directions. A schematic view of the model condition is shown in Figure S1.4.

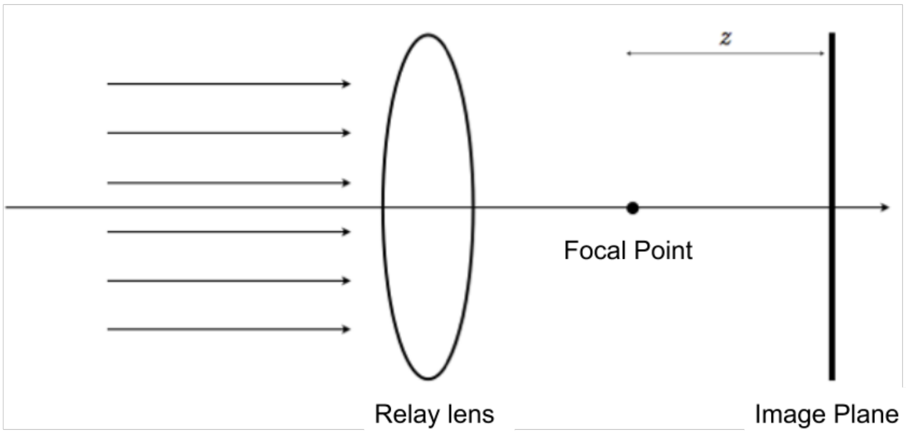

S1 Fig. S1.4: Boundary condition for Born-Wolf PSF model

Detection : The Monte Carlo simulation of a camera system includes a statistical model of a systematic source and generates digital images that closely represents the actual image obtained using an actual camera. We particularly simulate the detection process for CMOS and EMCCD cameras. Details of the camera simulations are described as follows.

(1) Uncertainty sources: Uncertainty sources of the camera systems are ruled by camera specifications and conditions shown in Table S1.2 [4, 5]. First, shot noise arises from statistical fluctuations in the number of photons incident to the camera. This noise source is a fundamental property of the quantum nature of light and is always present in imaging systems. The incident photons interact with the photodiode placed on a pixel plate. Photoelectric effects can convert the incident photon signals to photoelectrons. The probability for such a conversion is the so-called quantum efficiency (QE). As both photons and electrons are quantized, the detection process is characterized by binomial distributions. Finally, readout noise is generated whereas the photoelectron signals can be linearly digitized as an image in terms of the count 16-bit analog-to-digital converter (ADC). For CMOS and EMCCD cameras, the linear relationships of photoelectrons outputs with ADC outputs are shown in Figure S1.5.

n addition, the EMCCD camera has excess noise that increases the standard deviation of the output signal by $\sqrt{2}$ [32, 37, 38, 39]], whereas the CMOS and CCD cameras have no excess noise (1.0). The EMCCD camera uses the multiplication process, and each stage has a small gain to multiply the number of photoelectrons. Such process is stochastic and characterized by multistage binomial distributions, which increased noise.

Camera type	EMCCD	CMOS
Image size	100×100	100×100
QE	92 %	70 %
EM Gain	$\times$ 1, $\times$ 100, $\times$ 300	N/A
Exposure time	30 msec	30 msec
Readout noise	100 electrons	1.3 electrons
Excess noise	$\sqrt{2}$	1
A/D Converter	16 bit	16 bit
Gain	5.82 electrons/count	0.458 electrons/count
Offset	2000 counts	100 counts
Full well	370,000 electrons	30,000 electrons
Dynamic range	71.3 dB	87.2 dB

S1 Table S1.2: Camera specification and condition

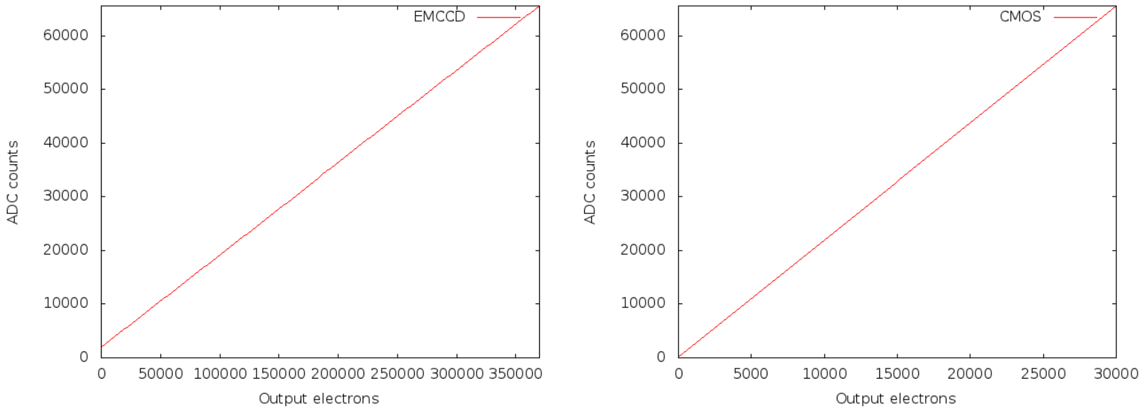

S1 Fig. S1.5: A/D converter linearity for EMCCD (left) and CMOS (right)

(2) Probability density function (PDF) per pixel: The camera pixel output is the convolution of the probability distributions of each of the systematic sources. The PDF of CMOS camera pixels is

given by the Poisson distribution and written in the form of

$$q(S_i|E_i) = \frac{E_i^{S_i} e^{-E_i}}{S_i!} \tag{10}$$

where S_i and E_i are the random number of output electrons and expectations in the i-th pixel, respectively. The left panel of Figure S1.6 shows the PDF with respect to the number of incident photons. The PDF of EMCCD camera pixels [32, 37] is written in the form of

$$q(S_i|E_i) = \exp(-E_i)\delta(S_i) + \sqrt{\frac{\alpha E_i}{S_i}} \exp(-\alpha S_i - E_i)\ I_1\left(2\sqrt{\alpha E_i S_i}\right) \tag{11}$$

where I_1 is the modified Bessel function of the first kind of order one. α is the inverse of the EM gain. Figure S1.7 and the right panel of Figure S1.6 show the PDF with respect to the number of incident photons.

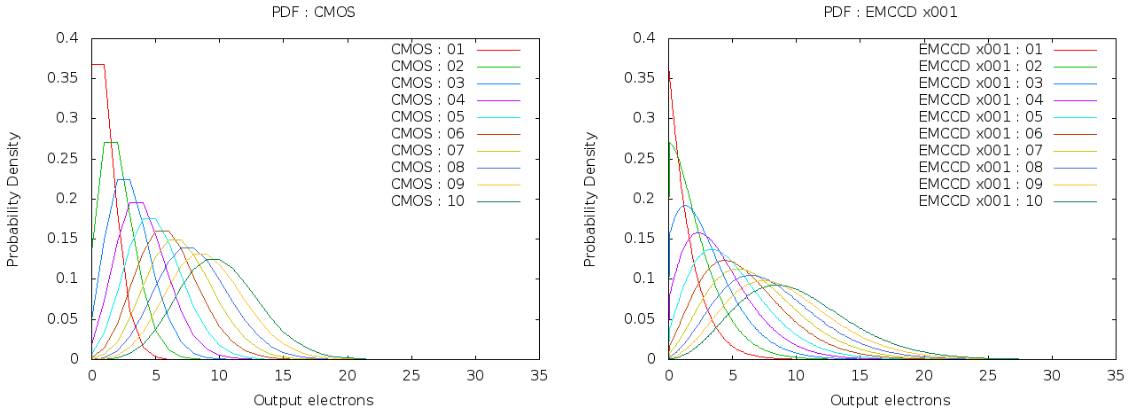

S1 Fig. S1.6: Probability density function for CMOS (left) and EMCCD (right)

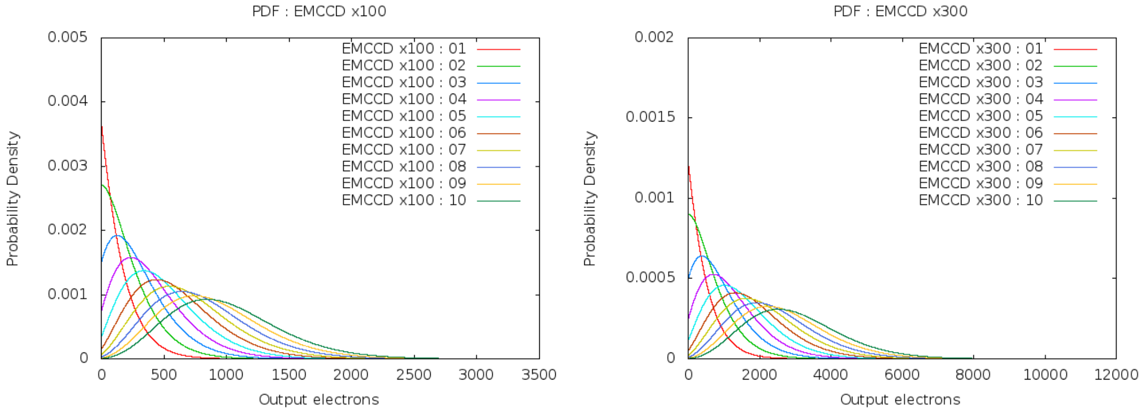

S1 Fig. S1.7: Probability density function for EMCCD : EM gain $\times 100$ (left) and $\times 300$ (right)

(3) Readout noise: Noise triggered by the readout electronics is typically dominated by the noise on the floating diffusion amplifier and the A/D converter. It increases with clocking speed or frame readout speed. This noise is the result of the statistical uncertainty that occurs when the amplifier attempted to reset itself to zero before the next image. The readout noise distribution for the EMCCD camera is usually Gaussian, as shown on the left panel of Figure S1.8 and Figure S1.9. However, the readout noise distribution for the CMOS camera is uneven, because of the differences in characteristics of the amplifiers in each pixel. The distribution is shown on the right panel of Figure S1.8 and Figure S1.10.

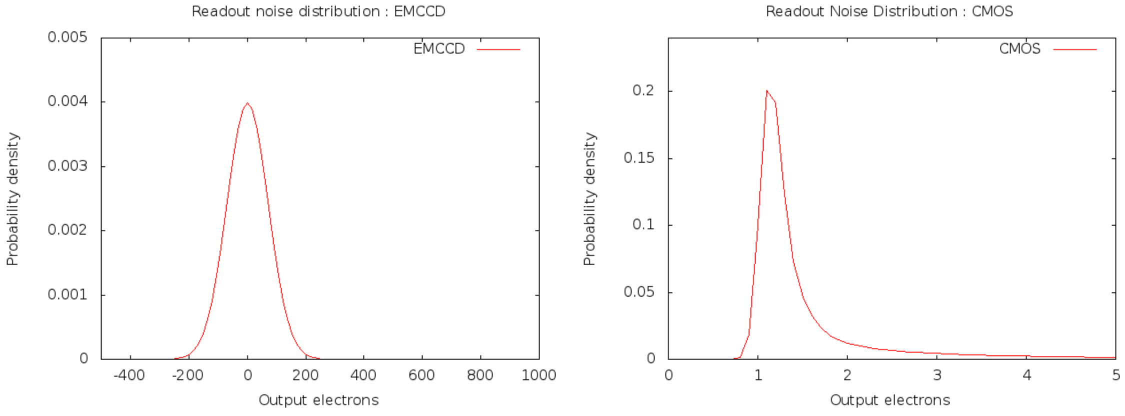

S1 Fig. S1.8: Readout noise distributions for EMCCD (left) and CMOS (right)

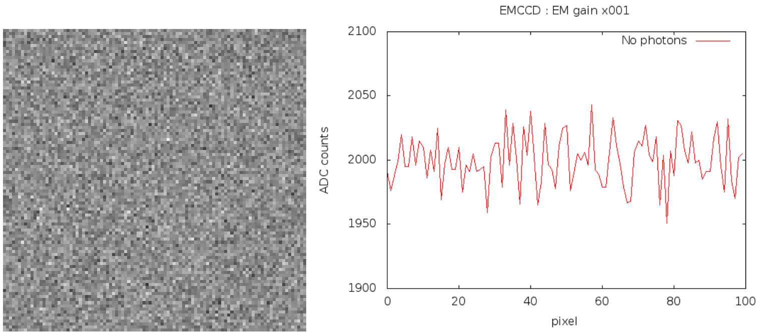

S1 Fig. S1.9: Readout noise for EMCCD. Image (left) and its intensity graph that depict the readout noise of horizontal line at vertical center (right)

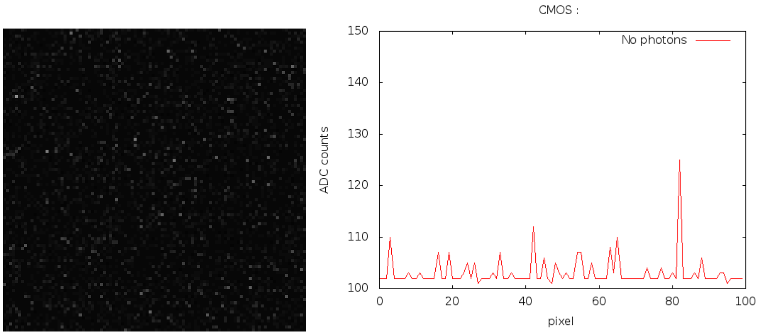

S1 Fig. S1.10: Readout noise for CMOS. Image (left) and its intensity graph that depict the readout noise of horizontal line at vertical center (right)

(4) SNR per pixel: The variance of the camera pixel output is given by the sum of the variances of all uncertainty sources. The SNR, which is the ratio of the output signal to the standard deviation of the signal, is written in the form of

$$SNR = \frac{QE \cdot S}{\sqrt{F_n^2 \cdot QE \cdot (S + I_b) + (N_r/M)^2}} \tag{12}$$

The input photon signal (S) and optical background (I_b) falling on the photodiodes have average photon flux per pixel. The fluctuations at this rate are governed by Poisson statistics and therefore have a standard deviation that is the square root of the number of photons ($\sqrt{S + I_b}$). The quantum efficiency (QE) of the camera is the wavelength dependent probability that photon

is converted to a photoelectron. Since the QE is predominated in the SNR equation, high QE is a fundamental attribute for obtaining high SNR. Readout noise (N_r) is a statistical expression of the variability within the electrons that convert the charge of the photoelectrons in each pixel to the number of ADC counts. EM gain (M) is a factor of electron multiplication. It occurs in voltage dependent, stepwise manner and the total amour is a combination of the voltage applied and number of steps in EM register. The EM gain also has a statistical distribution and an associated variance accounted for the excess noise factor (F_n). The SNR and relative SNR for three cameras specifications are shown in Figures S1.11 and S1.12.

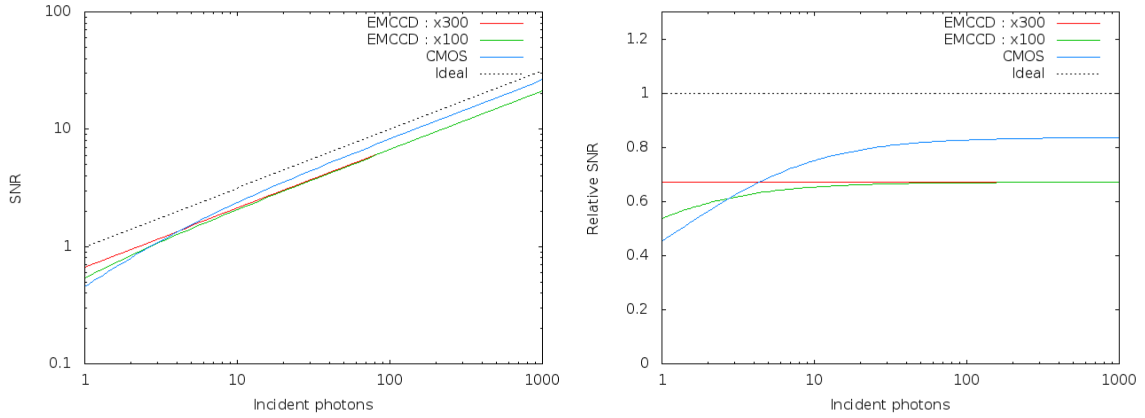

S1 Fig. S1.11: No background photons, SNR (left) and relative SNR (right) for CMOS and EMCCD

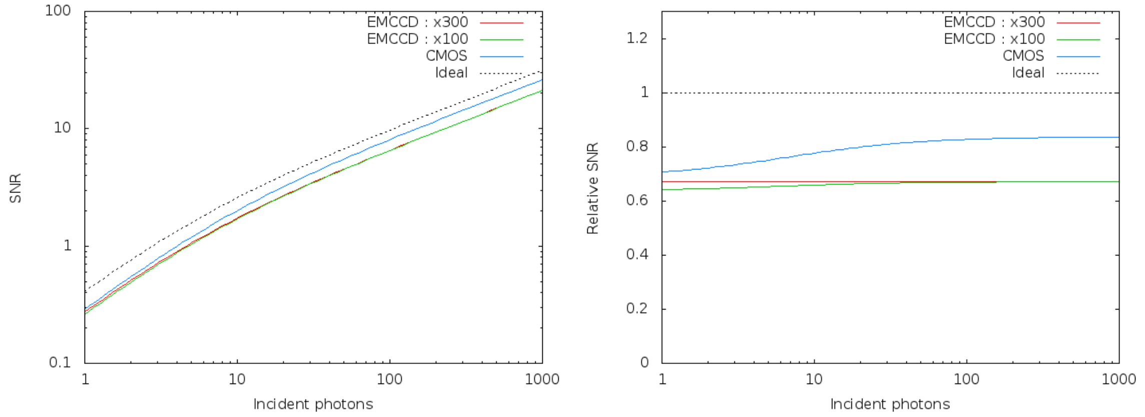

S1 Fig. S1.12: Five background photons, SNR (left) and relative SNR (right) for CMOS and EMCCD

1.1.4 Examples of images

Figure S1.13, S1.14 and S1.15 show images and their intensity graphs showing the signal intensity and noise of the horizontal line at the vertical center. From the top to bottom rows in each Figure, $1, 2, 4, 6, 8$, and 10 incident photons per pixel are expected in 80×80 pixel squares at the image center.

No background photon **5 background photons**

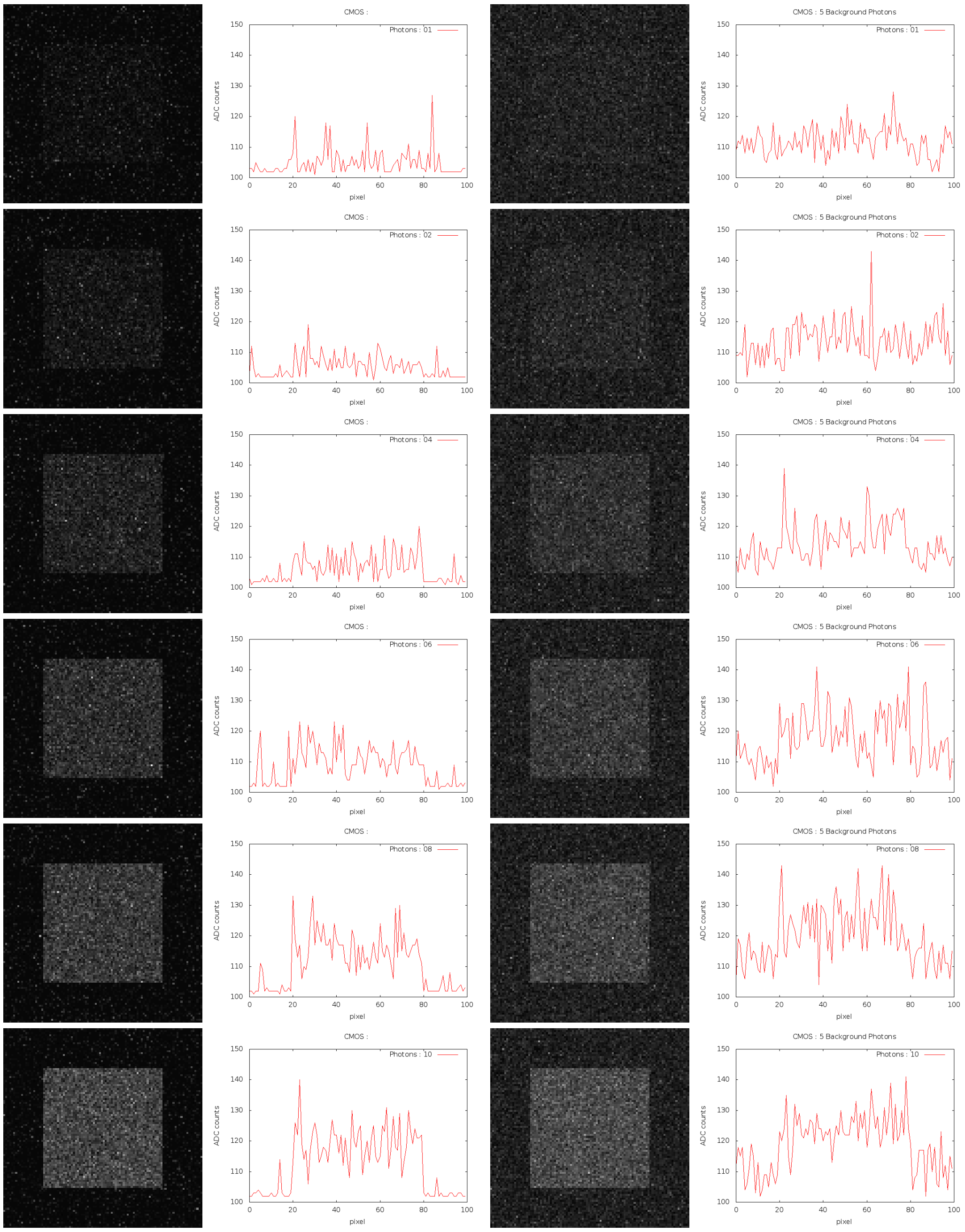

S1 Fig. S1.13: CMOS

No background photon **5 background photons**

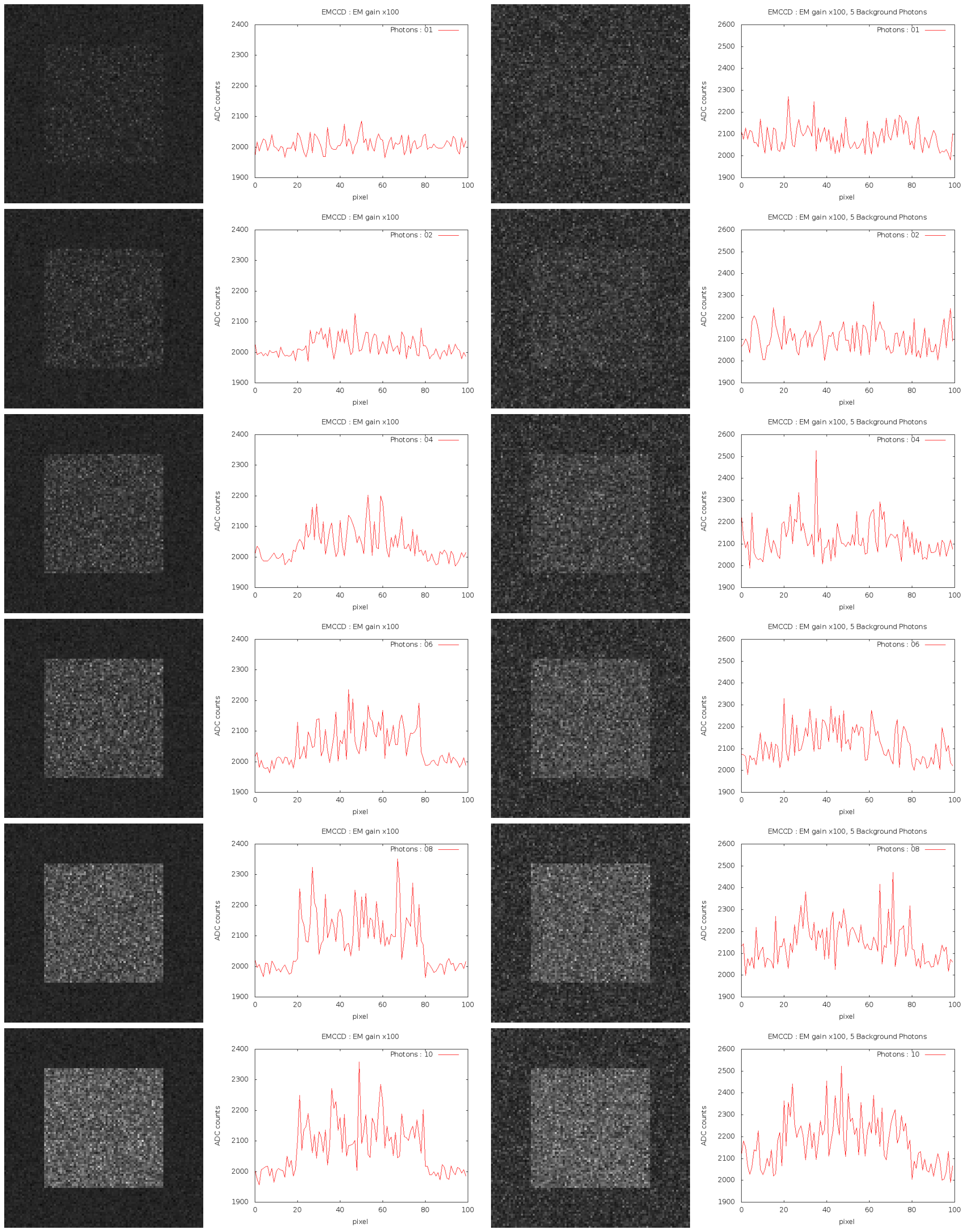

S1 Fig. S1.14: EMCCD EM gain ×100

No background photon **5 background photons**

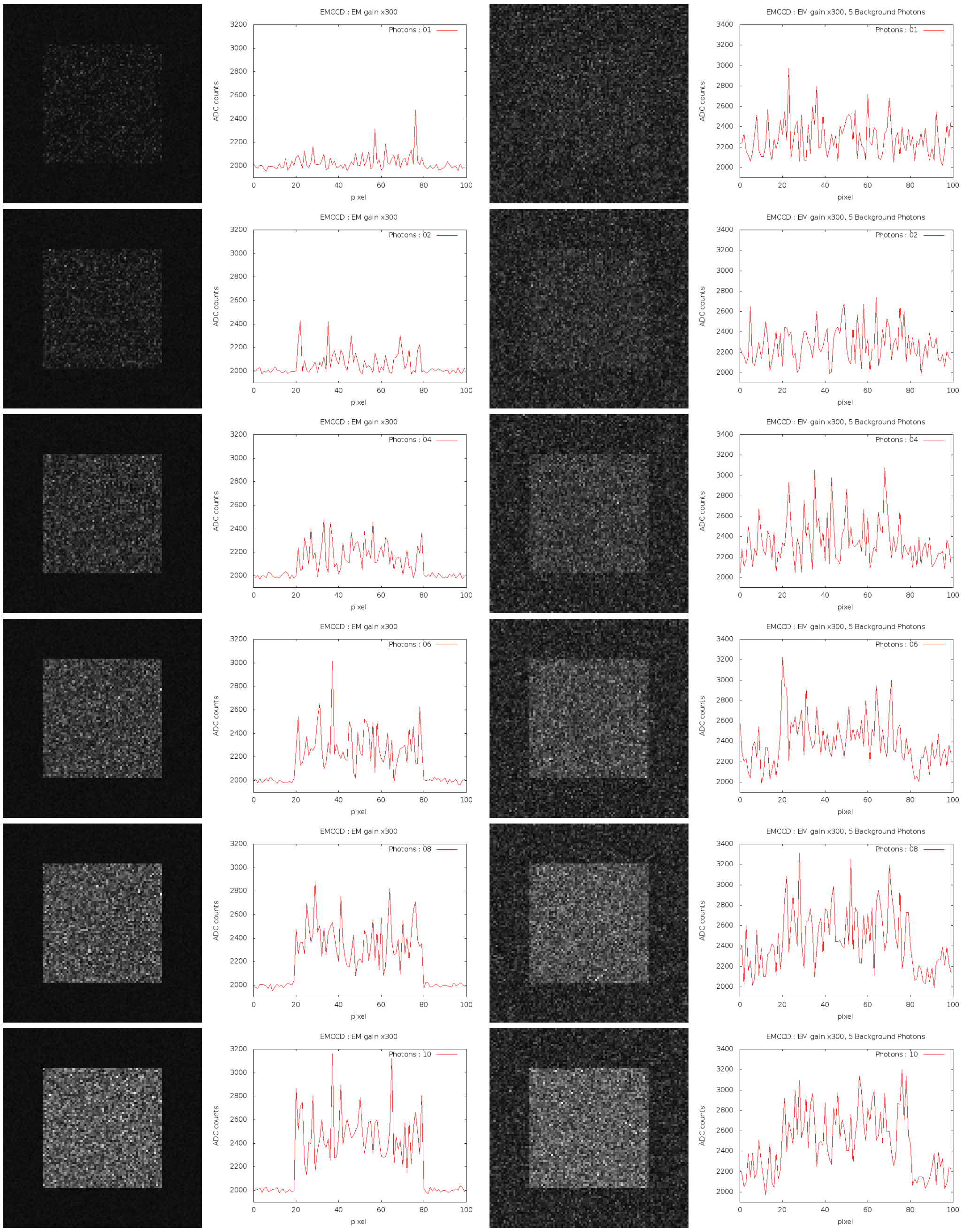

S1 Fig. S1.15: EMCCD EM gain ×300

Simple model 1 : We constructed relatively simple particle model of TMR on glass surface as shown in left of Figure S1.16. We assumed that 100 TMR molecules are stationary, and randomly distributed on the surface ($30 \times 30\ \mu m^2$). Images are simulated for the optical specification and condition of the TIRFM simulation module shown in Table S1.3. Right of Figure S1.16 is the expected image averaged by 160 images captured with CMOS camera. Results are shown in Figure S1.17. S1 Figs from top row to bottom one correspond to the beam inputs of $20, 30, 40$ and $50\ W/cm^2$, respectively.

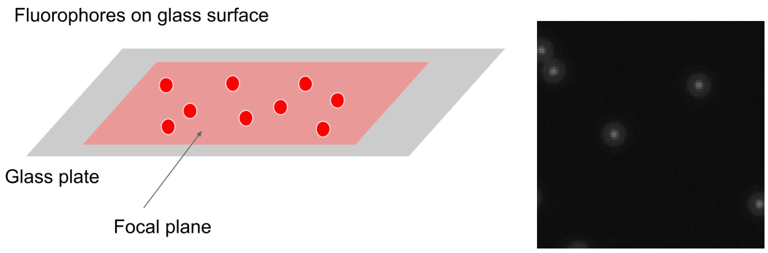

S1 Fig. S1.16: Fluorophores on glass surface (left) and the expected image (right).

Beam flux density	$20,\ 30,\ 40,\ 50\ W/cm^2$	
Beam wavelength	488 nm	
Refraction index	1.33 (glass), 1.27 (water)	
Critical angle	$65.6°$	
Fluorophore	TRITC (Abs. 548 nm/ Em. 608 nm)	
Objective	$\times$ 60 / N.A. 1.40	
Dichroic mirror	Semrok FF-562-Di03	
Emission filter	Semrok FF-593-25/40	
Linear conversion	10^{-6}	
Tube lens	$\times$ 4.2	$\times$ 1.67
Optical magnification	$\times$ 250	$\times$ 100
Camera type	EMCCD	CMOS
Image size	512×512	600×600
Pixel size	$16\ \mu m$	$6.5\ \mu m$
QE	92 %	70 %
EM Gain	$\times 300,\ \times 500$	N/A
Exposure time	30 msec	30 msec
Readout noise	100 electrons	1.3 electrons
Full well	370,000 electrons	30,000 electrons
Dynamic range	71.36 dB	87.2 dB
Excess noise	$\sqrt{2}$	1
A/D Converter	16-bit	16-bit
Gain	5.82 electrons/count	0.47 electrons/count
Offset	2000 counts	100 counts
Optical background	0.1 photons/pixel	

S1 Table S1.3: TIRFM specifications and condition to image the simple model 1.

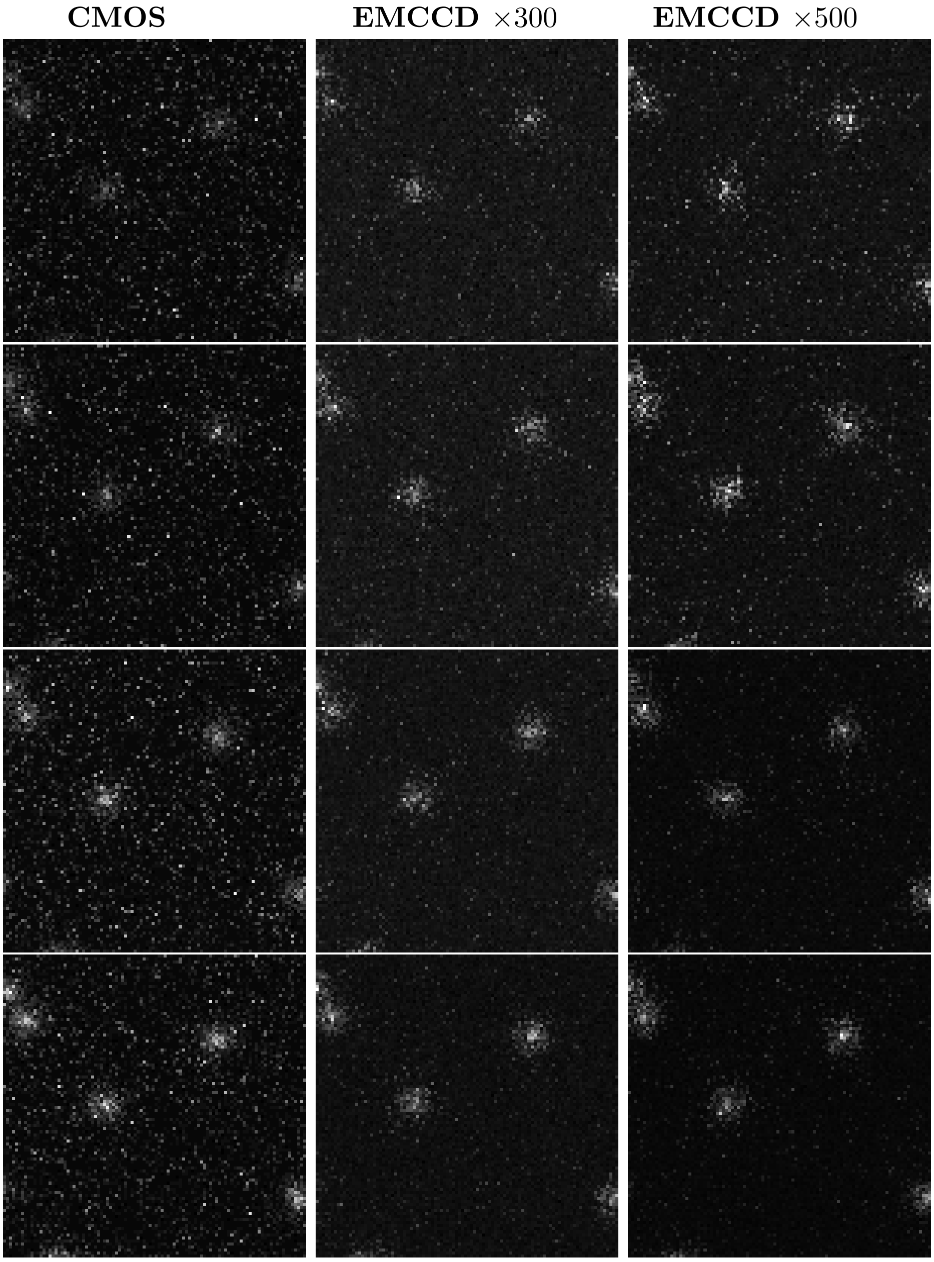

S1 Fig. S1.17: Comparison of single molecule images (100×100 pixels at image center). Increasing the beam flux density results in relatively smaller noise. Grayscale is the count number of 16-bit ADC.

Simple model 2 : Using the TIRFM simulation module, we simulated imaging the basal region of the simple cell model of TMR-tagged molecules diffusing on membrane and in cytoplasm. The images are simulated for the optical system and detector specification and conditions shown in Table S1.4. Results are shown in Figure S1.18. We assume the simple cell that express the molecules tagged with TMR fluorescent protein. Figure S1.18A and S1.18B show reaction and geometry of the model ($20 \times 20 \times 4$ μm^3), respectively. The model consists of 2 chemical species, 2 reactions and 4 kinetic parameters. 100 TMR-tagged molecules are distributed on the cell membrane and diffuse with 0.1 μm^2/sec. 2,000 TMR-tagged molecules are distributed in the cell cytoplasm and diffuse with 5.00 μm^2/sec. Association rate from the cytoplasm to the membrane of those molecule is 3.35 μm/sec. Dissociation rate from the membrane to the cytoplasm is 1.00 sec^{-1}.

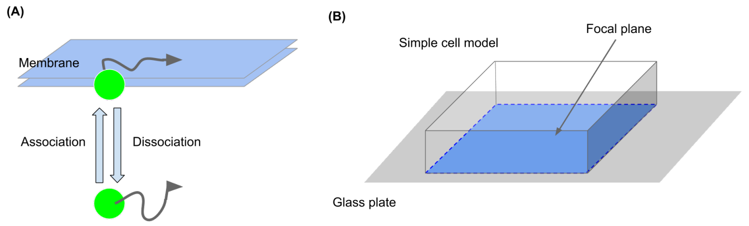

S1 Fig. S1.18: (A) schematics of network and (B) geometry of the simple cell model.

Beam flux density	40 W/cm^2	
Beam wavelength	488 nm	
Refraction index	1.33 (glass), 1.27 (water)	
Critical angle	65.6°	
Fluorophore	TRITC (Abs. 548 nm/ Em. 608 nm)	
Objective	× 60 / N.A. 1.40	
Dichroic mirror	Semrok FF-562-Di03	
Emission filter	Semrok FF-593-25/40	
Linear conversion	10^{-6}	
Tube lens	× 4.2	× 1.67
Optical magnification	× 250	× 100
Camera type	EMCCD	CMOS
Image size	512 × 512	600 × 600
Pixel size	16 μm	6.5 μm
QE	92 %	70 %
EM Gain	×300	N/A
Exposure time	30 msec	30 msec
Readout noise	100 electrons	1.3 electrons
Full well	370,000 electrons	30,000 electrons
Dynamic range	71.36 dB	87.2 dB
Excess noise	$\sqrt{2}$	1
A/D Converter	16-bit	16-bit
Gain	5.82 electrons/count	0.47 electrons/count
Offset	2000 counts	100 counts
Optical background	0.1 photons/pixel	

S1 Table S1.4: TIRFM specifications and condition to image the simple model 2.

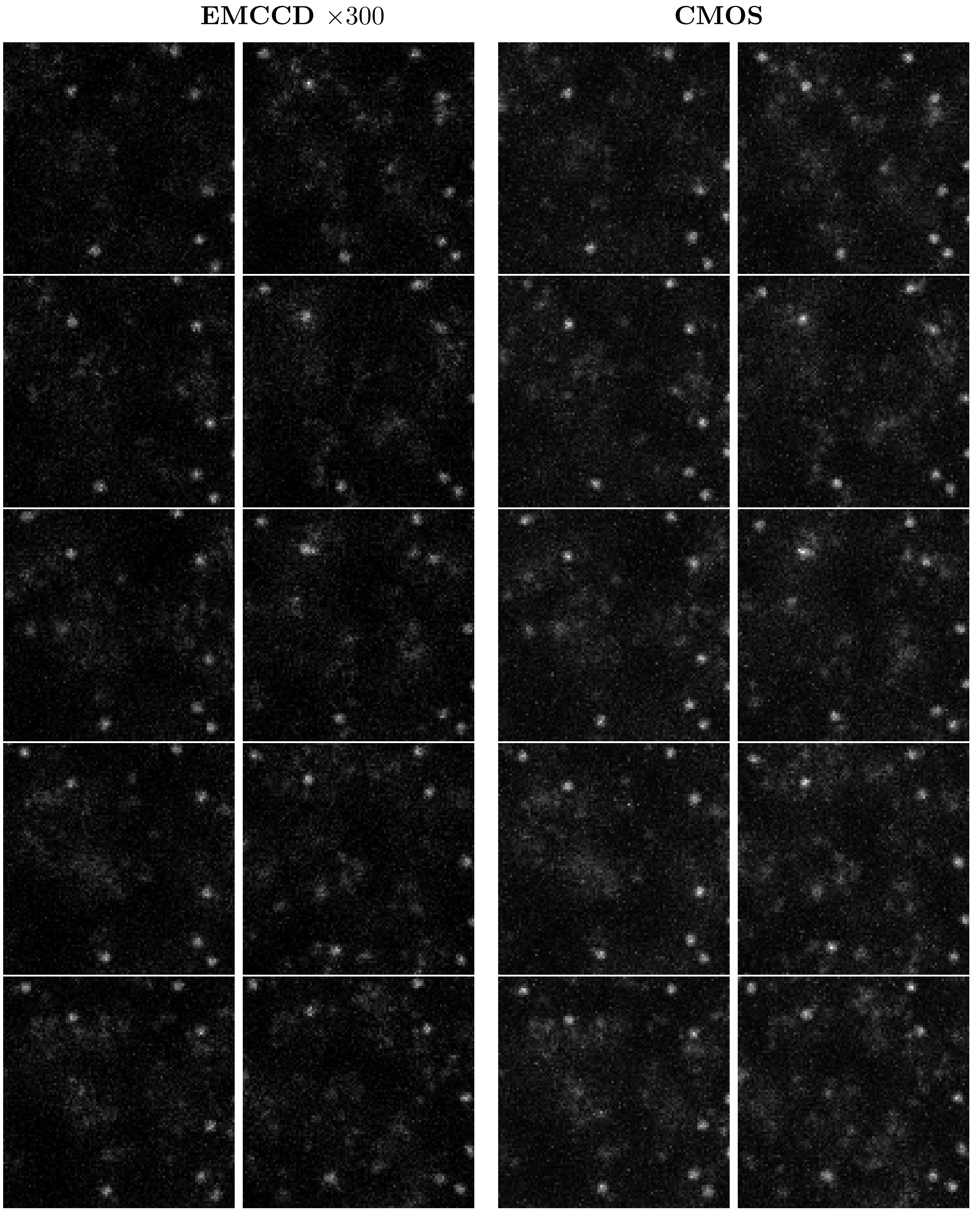

S1 Fig. S1.19: Example images of continuous 10 frames (100 × 100 pixels).

Simple model 3 : Using the TIRFM simulation module, we simulated imaging the basal region of the two state model of TMR-tagged molecules diffusing on membrane. The images are simulated for the optical system and detector specification and conditions shown in Table S1.5. Results are shown in Figure S1.20. We assume the simple cell that express the molecules tagged with TMR fluorescent protein. Figure S1.20 shows geometry of the two state model ($20 \times 20 \times 4\ \mu\mathrm{m}^3$). The model consists of 1 chemical species and 2 kinetic parameters. 200 TMR-tagged molecules are distributed on the cell membrane and fast diffuse with 0.2 $\mu\mathrm{m}^2$/sec. 300 TMR-tagged molecules are distributed on the cell membrane and slow diffuse with 0.02 $\mu\mathrm{m}^2$/sec.

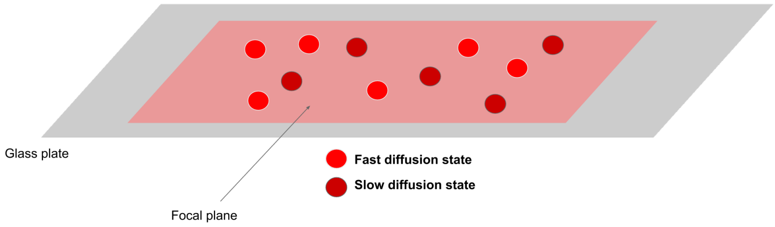

S1 Fig. S1.20: geometry of the two state model.

Beam flux density	10 W/cm^2	
Beam wavelength	488 nm	
Refraction index	1.33 (glass), 1.27 (water)	
Critical angle	65.6°	
Fluorophore	TRITC (Abs. 548 nm/ Em. 608 nm)	
Objective	$\times$ 60 / N.A. 1.40	
Dichroic mirror	Semrok FF-562-Di03	
Emission filter	Semrok FF-593-25/40	
Linear conversion	10^{-6}	
Tube lens	$\times$ 4.2	$\times$ 1.67
Optical magnification	$\times$ 250	$\times$ 100
Camera type	EMCCD	CMOS
Image size	512×512	600×600
Pixel size	16 μm	6.5 μm
QE	92 %	70 %
EM Gain	$\times$300	N/A
Exposure time	30 msec	30 msec
Readout noise	100 electrons	1.3 electrons
Full well	370,000 electrons	30,000 electrons
Dynamic range	71.36 dB	87.2 dB
Excess noise	$\sqrt{2}$	1
A/D Converter	16-bit	16-bit
Gain	5.82 electrons/count	0.47 electrons/count
Offset	2000 counts	100 counts
Optical background	0.1 photons/pixel	

S1 Table S1.5: TIRFM specifications and condition to image the two state model.

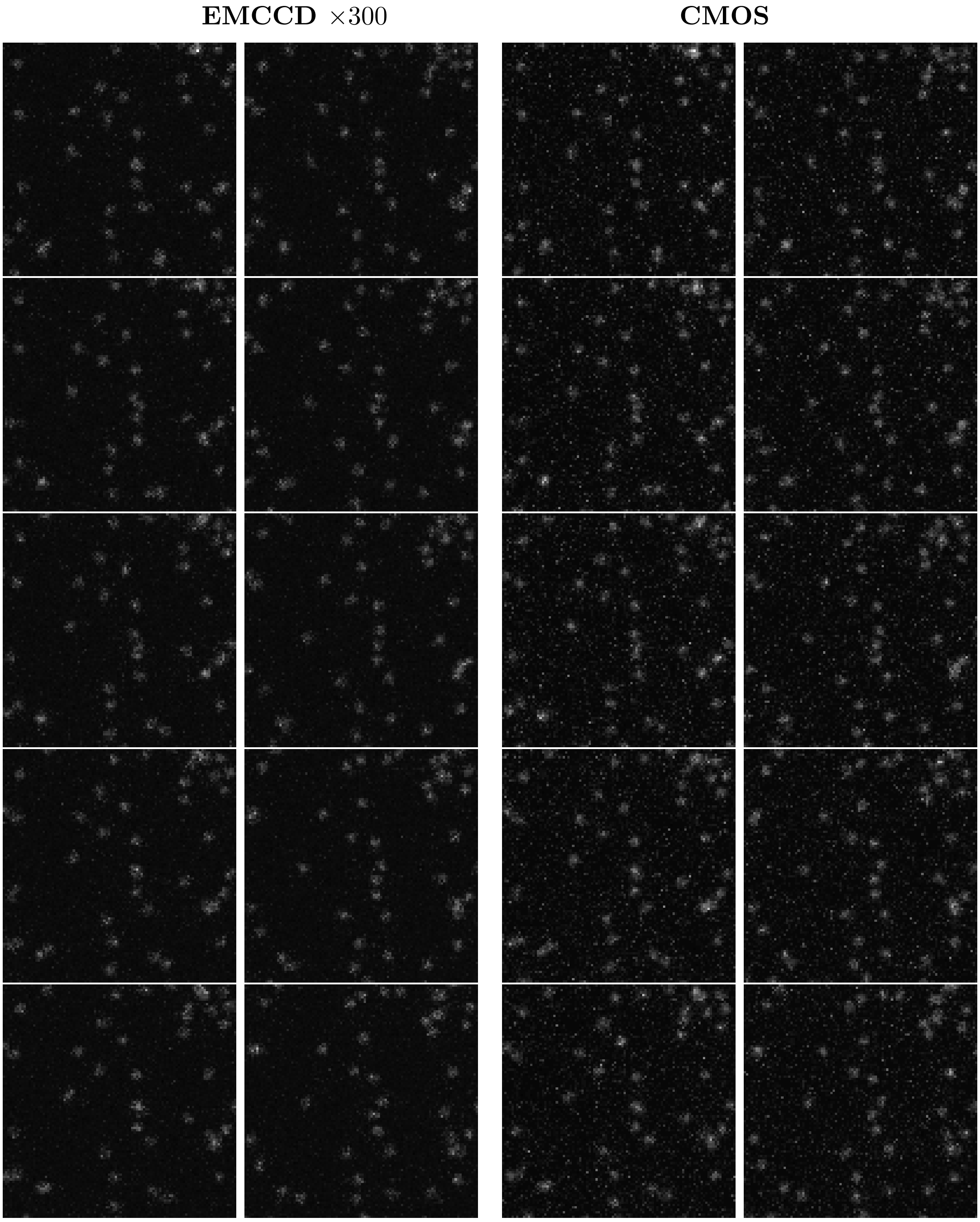

S1 Fig. S1.21: Example images of continuous 10 frames (100 × 100 pixels).

1.2 LSCM simulation module

The LSCM simulation module enables a selective visualization of focal regions of cell model. Its optical configuration is shown in Figure S1.22. Implementation assumptions are summarized in Table S1.6.

Principle	Photon-counting<br>1st-order paraxial approximation (Linear term)
Illumination	Gaussian beam profile<br>Continuous / Gaussian / Unpolarized
Fluorescence	Linear convertion ($\times 10^{-6}$)<br>Cross-section ($\sigma \cong 10^{-14}$ cm^2)
Image-forming	3-D Airy PSF (Unpolarized form)<br>PMT

S1 Table S1.6: Implementation assumption for the LSCM simulation module. Detection process for the PMT is performed with Monte Carlo simulation.

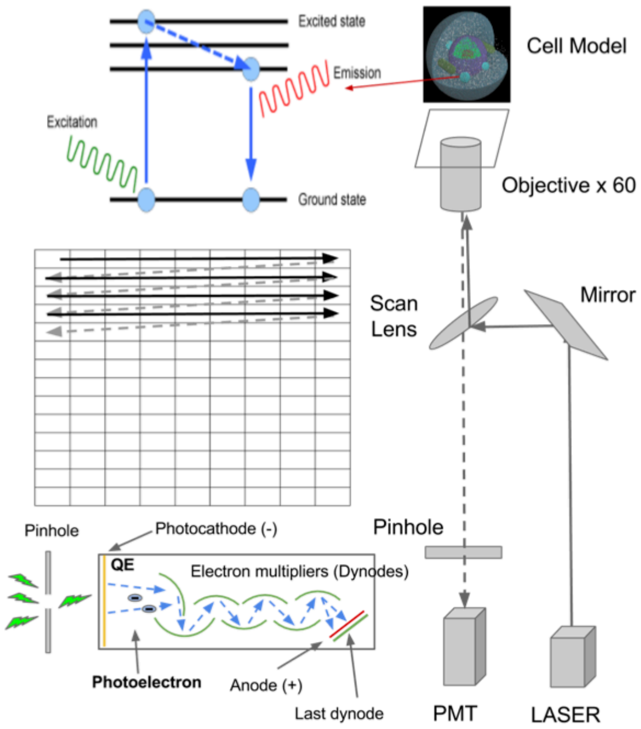

S1 Fig. S1.22: Optical configurations of the LSCM simulation module

1.2.1 Illumination system

Uncertainty sources of the illumination system are ruled by specification and conditions of Gaussian beam profile. We assume ideal Gaussian laser-beam intensity profile, which corresponds to the theoretical TEM_{00} mode. The Gaussian beam wavefront of excitation wavelength continuously illuminate the specimen, and propagate perfectly flat with all elements moving precisely in the parallel direction. The wavefront quickly generate the $1/e^2$ irradiance contour at the plane after the wavefront

has propagated a distance z. The contour spreads in the form of

$$w(z) \;=\; w_0\sqrt{1+\left(\frac{\lambda z}{\pi w_0^2}\right)^2} \tag{13}$$

where w_0 is the beam waist radius at the focal plane where the wavefront is assumed to be flat. z is the distance of propagation from the focal plane where the wavefront is assumed to be flat. λ is the wavelength of excitation. The intensity of the Gaussian TEM_{00} beam is written in the form of

$$I(r,z) \;=\; \frac{2P'}{\pi w(z)^2}\exp\left(-2r^2/w(z)^2\right) \tag{14}$$

where P' is the beam flux at the level of photon-counting unit [#photons/sec]. More details are described in ref. [17, 43].

1.2.2 Image-forming system

The Monte Carlo simulation for the photomultipliers tube (PMT) includes a statistical model of noise source. Emitted photons of longer wavelengths are distributed as the sum of the PSFs shown in Eq. (9). As the incident beam is scanned across the cell model in horizontal and vertical axes, a digital image that closely represents the actual confocal image can be obtained pixel-by-pixel. Details of the PMT simulation are described as follows.

(1) Uncertainty sources [17, 44]: Uncertainty sources of the PMT system are ruled by PMT specifications and conditions shown in Table S4. First, shot noise arises in the number of photons incident to the PMT. When the incident photons interact with the photocathode placed on the head part of a PMT, photoelectrons are emitted. These photoelectrons are multiplied by the cascade process of secondary emission through a series of dynodes and finally reach the anode connected to an output processing circuit. The methods of readout processing the output signal of a PMT can be broadly divided into the analog and digital (photon counting) modes, depending on the number of incident photons and the bandwidth of the output processing circuit. If the output pulse-to-pulse interval is narrower than each pulse width or if the signal processing circuit is not sufficiently fast, then the actual output pulses overlap and become a direct current with shot noise fluctuations. This method is in the analog mode. In contrast, if the output pulse intervals are separated from noise pulses, discrete output pulses can then be detected by the photon counting method.

PMT mode	Photon-counting	Analog
QE	30 %	
Dynode	11 stages	
Average gain	$\times 10^6$	
Readout noise	0 counts/sec	0 mA
Excess noise	N/A	1.1
Pair-pulse time	18 nsec	N/A
Optical background	0.00 photons/sec	

S1 Table S1.7: PMT specifications and condition.

(2) Probability density function (PDF) [45] : An approximate PDF at the output of the PMT is written in the form of

$$q(S|E) \;=\; e^{(E(e^{-A/B})-1)}\delta(S) + \frac{\sqrt{A/S}e^{-(E+S/B)}}{B}\sum_{n=0}^{\infty}\frac{\sqrt{n}(Ee^{-A/B})^n}{n!}I_1\left(\frac{2\sqrt{nAS}}{B}\right) \quad (15)$$

where I_1 is the modified Bessel function of the first kind of order one. The PMT is characterised by its average gain A and the number of dynode stages ν. The variance of the PMT output is $2AB$ where $B = 1/2(A-1)/(A^{1/\nu}-1)$. Assuming $A = 10^6$ and $\nu = 11$. E is the number of photoelectrons emitted at photocathode (expectation). Approximated formulas are described in ref. [46]. The PDFs in the photon-counting mode and analog mode are shown in Figure S1.23.

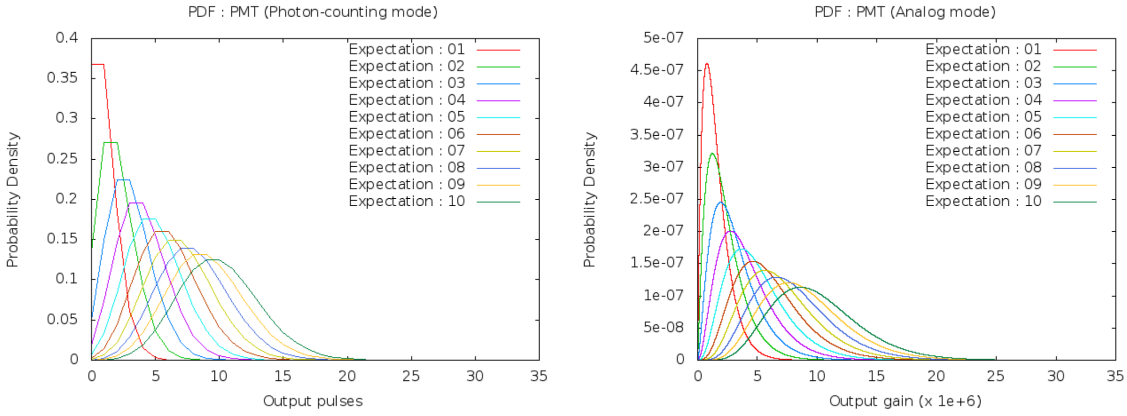

S1 Fig. S1.23: Probability density function for photon-counting mode (left) and analog mode (right)

(3) Count rate and linearity [44]: The photon counting mode offers excellent linearity over a wide range. The lower limit is determined by the number of dark current pulses. Maximum count rate is limited by the pair-pulse time resolution where two pulses can be separated at a minimum time interval. The measured count rate is given as

$$M = \frac{N_s}{1 + N_s\delta t} \quad (16)$$

where N_s is the input photon flux, and δt is the pair-pulses time resolution ($\sim$ 18 nsec). Linearity is shown in Figure S1.24.

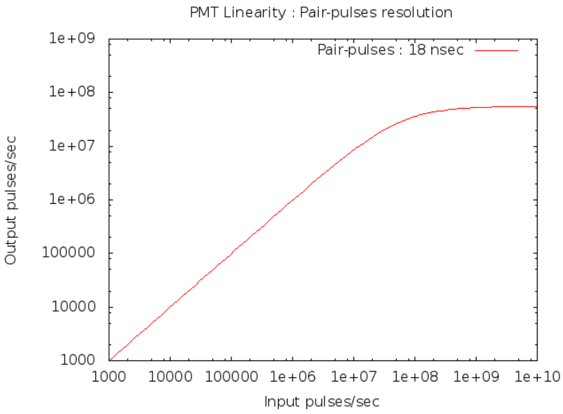

S1 Fig. S1.24: Linearity for photon-counting mode

(3) SNR per pixel [44] : The variance of the PMT output is given by the sum of the variances of all noise sources. The SNR and detection limits are plotted in Figure S1.25. The SNR in the analog mode is written in the form of

$$SNR = \frac{I_k}{\sqrt{2eBF\left(I_k + 2\left(I_d + I_b\right)\right) + \left(N_A/G\right)^2}} \tag{17}$$

where $I_k = eQEN_p$ and N_p is the number of incident photons/sec. I_d is dark current. B is bandwidth in Hz ($B = 1/(2T)$) and T is the observational period. G is gain factor ($\sim 10^6$). F is the excess noise ($F \approx \delta/(\delta - 1)$) and δ is the number of dynode stages. Detection limits ($SNR = 1$) as a function of bandwidth is given by the following equation.

$$N_p^{limit} = \frac{eBF + \sqrt{(eBF)^2 + (4eBFI_d)}}{eQE} \tag{18}$$

The SNR in the photon-counting mode is written in the form of

$$SNR = \frac{N_s}{\sqrt{\left(N_s + 2\left(N_d + N_b\right)\right)/T + N_A^2}} \tag{19}$$

where $N_s = QE \cdot N_p$ and N_d is the dark count/sec. The detection limit is also given by the following equation.

$$N_p^{limit} = \frac{B + \sqrt{B^2 + 4BN_d}}{QE} \tag{20}$$

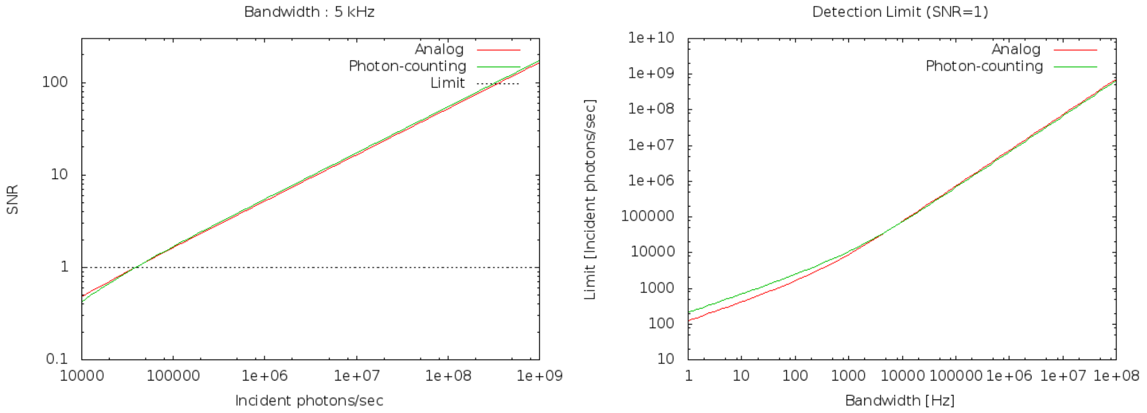

S1 Fig. S1.25: SNR (left) and detection limit when $SNR = 1$ (right)

1.2.3 Examples of outputs

Despite the absence of the scanning process, Figure S1.26 and S1.27 show images of outputs and the graphs showing the signal intensity and noise of the horizontal line at vertical center. From the top to bottom rows in each figure, $10^3, 10^4, 10^5, 10^6$, and 10^7 incident photon fluxes are expected in 80×80 pixel squares at the image center.

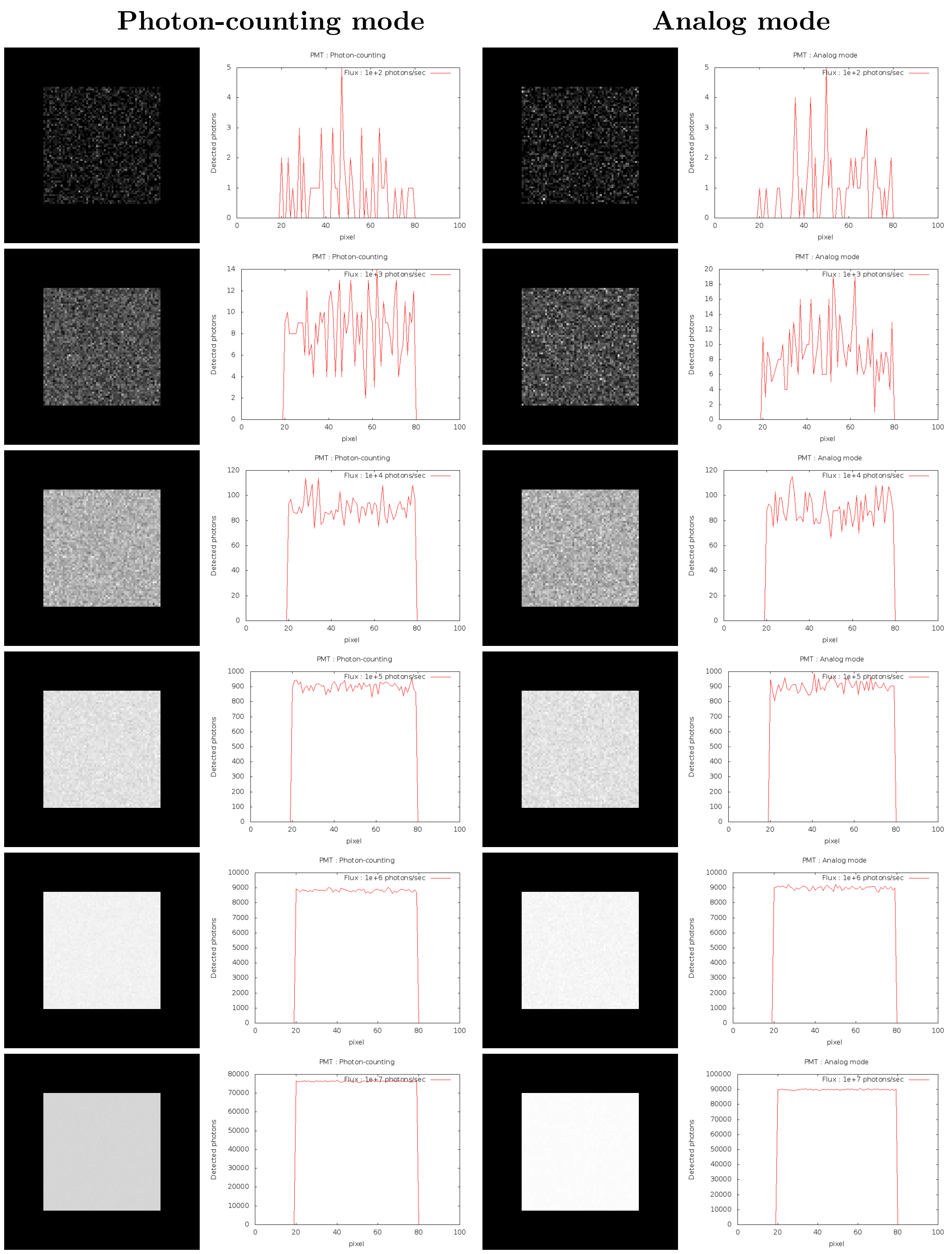

S1 Fig. S1.26: Image output of PMT. No dark count rate

Photon-counting mode **Analog mode**

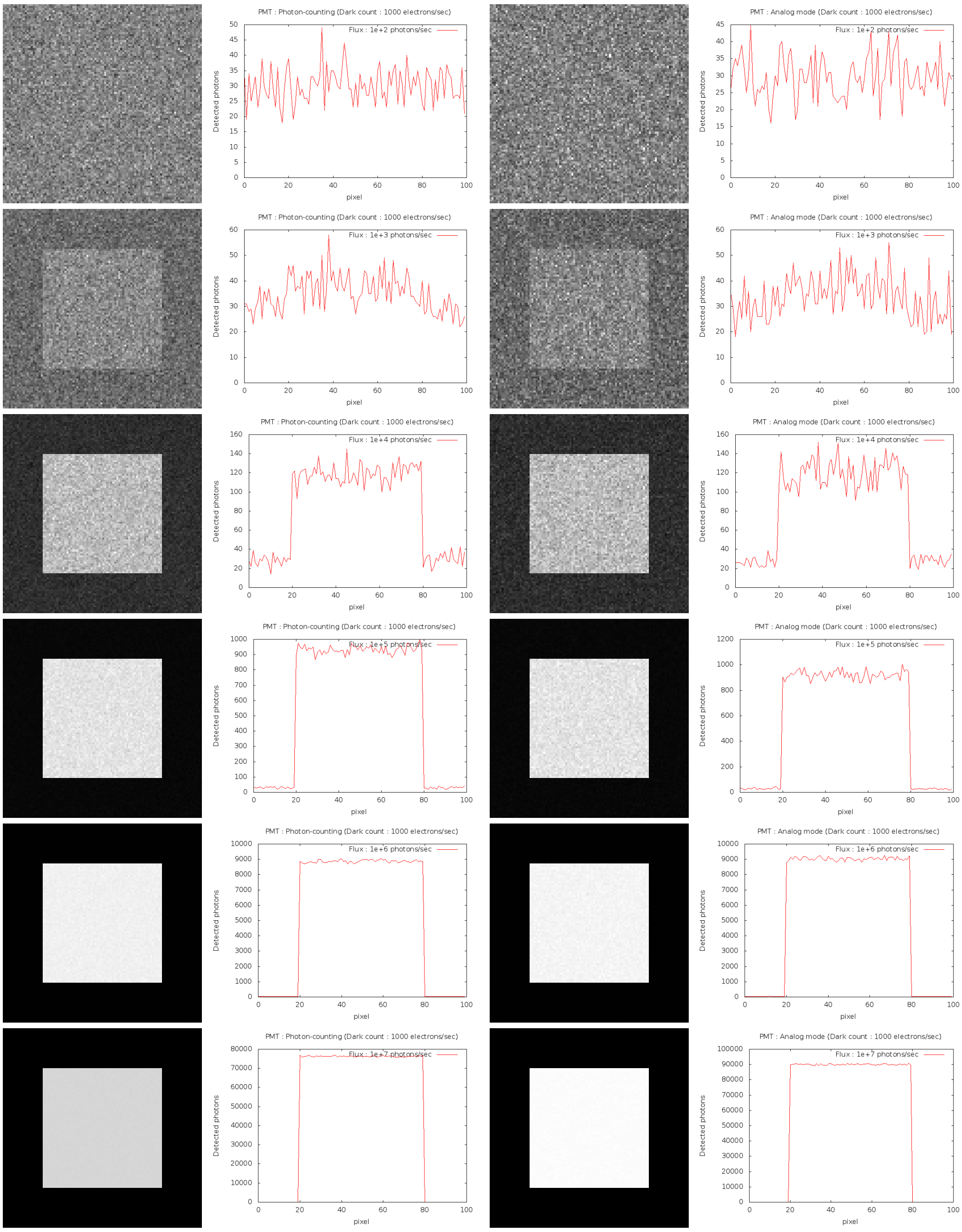

S1 Fig. S1.27: Image output of PMT. Background count rate is 1000 electrons/sec.

Simple model : We constructed relatively simple particle model of TMR in aqueous solution as shown in Figure S1.28. We assumed that $19,656$ TMR molecules are distributed in the solution box $(30 \times 30 \times 6\ \mu m^3)$, and diffuse with 100 μm^2/sec. Images are simulated for the optical specification and condition of the LSCM simulation module shown in Table S1.8. Results are shown in Figure S1.29. Figures from top row to bottom one correspond to the beam inputs of $10, 30, 70, 100$ and 300 μW, respectively.

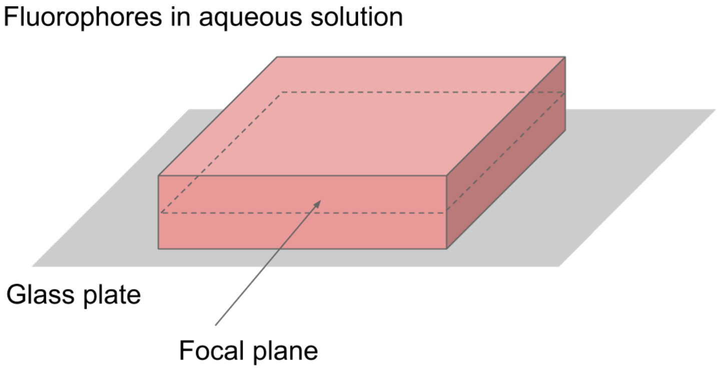

S1 Fig. S1.28: Fluorophores in aqueous solution.

Beam flux	30, 70, 100, 300 μW	
Beam wavelength	488 nm	
Beam waist	200 nm (Assumed)	
Fluorophore	mEGFP (Abs. 484 nm/ Em. 507 nm)	
Objective	$\times$ 60 / N.A. 1.49	
Scan lens	$\times$ 1	
Pinhole	57.6 μm diameter (2 A.U)	
Optical magnification	$\times$ 60	
Linear conversion	10^{-6}	
Scan time	1.1 μsec/pixel	
Pixel length	210 nm/pixel	
Image size	1024×1024	
PMT mode	Photon-counting	Analog
A/D Converter	12-bit	12-bit
QE	30 %	30 %
Readout noise	0 counts/sec	0 mA
Excess noise	N/A	1.1
Optical background	0.10 photons/sec	

S1 Table S1.8: LSCM specifications and condition to image the simple particle model of fluorescent molecules.

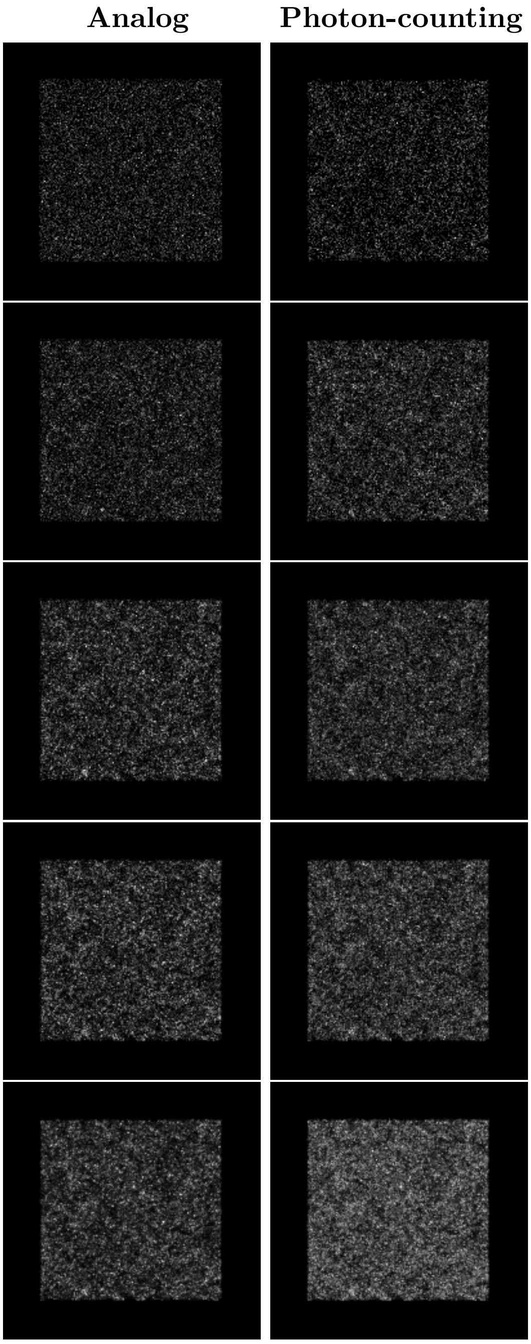

S1 Fig. S1.29: Image comparison (200 × 200 pixels at image center)

References

[1] Bevington, P. R. and Robinson, D. K. (2003) Data Reduction and Error Analysis. 3rd ed. McGrawHill

[2] Taylor, J. R. (1997) An Introduction to Error Analysis. 2nd ed. University Science Book

[3] Huang, F. et al. (2013) Video-rate nanoscopy using sCMOS camera-specific single-molecule localization algorithms. Nat. Methods, 10, 653-658.

[4] Fullerton, S. M. *et al.* (2012) "Camera Simulation Engine Enables Efficient System Optimization for Super-Resolution Imaging." *Proc. SPIE* 8228, Single Molecule Spectroscopy and Superresolution Imaging V, 822811; doi:10.1117/12.906346

[5] Fullerton, S. M. *et al.* (2012) "Optimization of Precise Localization Microscopy using CMOS Camera Technology." *Proc. SPIE* 8228, Single Molecule Spectroscopy and Superresolution Imaging V, 82280T; doi:10.1117/12.906336

[6] Sornette, D. *et al.* (2007) Algorithm for model validation: theory and applications. Proc. Natl. Acad. Sci. U. S. A., 104, 6562-7.

[7] Trucano,T.G. et al. (2006) Calibration, validation, and sensitivity analysis: What's what. Reliab. Eng. Syst. Saf., 91, 1331-1357.

[8] Oberkampf, W. L. *et al.* (2004) Verification, validation, and predictive capability in computational engineering and physics. Appl. Mech. Rev., 57, 345.

[9] Takahashi,K. et al. (2005) Space in theoretical biology of signaling pathways-towards intracellular molecular crowding in silico. FEBS Lett., 579, 1783-1788.

[10] Van Zon, J. S. and ten Wolde, P. R. (2005) Green's-function reaction dynamics: a particle-based approach for simulating biochemical networks in time and space. J. Chem. Phys., 123, 234910.

[11] Takahashi, K., Tanase-Nicola, S. and ten Wolde, P. R. (2010) Spatio-temporal correlations can drastically change the response of a MAPK pathway. *Proc Natl Acad Sci* USA 107, 2473-2478.

[12] Arjunan, S. N. V., and Tomita, M., (2010) A new multicompartmental reaction-diffusion modeling method links transient membrane attachment of *E. coli* MinE to E-ring formation, *Syst. Symth. Biol.*, 10.1007/s11693-009-9047-2

[13] S.S. Andrews, D. Bray, (2004) Stochastic simulation of chemical reactions with spatial resolution and single molecule detail, Phys. Biol. 1 3:137

[14] R.A. Kerr, et al., (2008) Fast Monte Carlo simulation methods for biological reactiondiffusion systems in solution and on surfaces, SIAM J. Sci. Comput. 30 6:31263149

[15] I.I. Moraru, et al., (2008) Virtual cell modelling and simulation software environment, IET Syst. Biol. 2 5:352362

[16] Mansuripur, M. (2009) Classical optics and its application, 2nd edition

[17] Pawley, J., (2008) Handbook of Biological confocal microscopy *Springer*, 3rd ed.

[18] Valeur, B. and Berberan-Santos, M. N. (2012) Molecular fluorescence, *WILEY-VCH*, 2nd edition

[19] Lakowicz, J. R. (2006) Principles of fluorescence spectroscopy, *Springer*, 3rd edition

[20] Gaskill, J. D. (1987) Linear Systems, Fourier Transforms, and Optics, John Wiley & Sons, Inc., New York.

[21] Arjunan, S. N. V., and Tomita, M., (2009) Modeling reaction-diffusion of molecules on surface and in volume spaces with the E-Cell System. *Int. J. Comp. Sci. Info. Sec.* 3(1): 211-216.

[22] Kirshner,H. et al. (2013) 3-D PSF fitting for fluorescence microscopy: Implementation and localization application. J. Microsc., 249, 13–25.

[23] `http://bioimaging.jp/learn/018/`

[24] Szekely,T. and Burrage,K. (2014) Stochastic simulation in systems biology. Comput. Struct. Biotechnol. J., 12, 14–25.

[25] Cvijovic, M. *et al.* (2014) Bridging the gaps in theoretical biology. Mol. Genet. Genomics.

[26] Qu, Z. et al. (2011) Multi-scale modeling in biology: how to bridge the gaps between scales? Prog. Biophys. Mol. Biol., 107, 21-31.

[27] Kitano, H. (2002) Theoretical biology: a brief overview. Science, 295, 1662-4.

[28] Kitano,H. (2002) Computational theoretical biology. Nature, 420, 206-10.

[29] Sanghvi, J. C. et al. Accelerated discovery via a whole-cell model. Nat. Methods 10, 1192-5 (2013).

[30] Karr, J. R. *et al.* (2012) A whole-cell computational model predicts phenotype from genotype. Cell, 150, 389-401.

[31] Tomita, M. (2001) Whole-cell simulation: a grand challenge of the 21st century. Trends Biotechnol., 19, 205-10.

[32] Mortensen, K. I. et al. (2010) Optimized localization analysis for single-molecule tracking and super-resolution microscopy. Nat. Methods, 7, 377-81.

[33] Miyanaga, Y,. Matsuoka, S. and Ueda, M., (2009) Single-Molecule Imaging Techniques to Visualize Chemotactic Signaling Events on the Membrane of Living Dictyostelium Cells *Methods in Molecular Biology*, 571, 417-435

[34] Axelrod, D. (2008) Chapter 7: Total internal reflection fluorescence microscopy. 1st ed. Elsevier Inc.

[35] Wazawa, T., Ueda, M., and Rietdorf, J. (2005) Microscopy Techniques, *Springer* 1297-1300

[36] Axelrod, D. (2003) Total internal reflection fluorescence microscopy in cell biology. Methods Enzymol., 361, 1-33.

[37] Hirsch,M. et al. (2013) A stochastic model for electron multiplication charge-coupled devices–from theory to practice. PLoS One, 8, e53671.

[38] EMImag technical guide, Hamamatsu Photonics, `http://www.hamamatsu.com/resources/pdf/sys/e_imagemtec.pdf`

[39] Robbins, M. S. et al. (2003) The Noise Performance of Electron Multiplying Charge-Coupled Devices. 50, 1227-1232.

[40] ORCA-Flash 2.8 technical guide, Hamamatsu Photonics, `http://www.hamamatsu.com/resources/pdf/sys/e_flashtec.pdf`

[41] Fullerton, S. M. *et al.* (2011) "Changing the Game." Hamamatsu Coorperation, http://sales.hamamatsu.com/assets/pdf/hpspdf/e_flash4.pdf

[42] Brankner, S. and Hobson, M. (2013) Synchronization and Triggering with the ORCA-Flash4.0 Scientific CMOS Camera, Hamamatsu Photonics, http://www.hamamatsu.com/resources/pdf/sys/SCAS0098E01_synchronization.pdf

[43] Introduction to Gaussian beam optics, CVI Melles Griot, http://www.physast.uga.edu/files/phys3330_fertig/Gaussian-Beam-Optics.pdf

[44] PHOTOMULTIPLIER TUBES Basics and Applications, Hamamatsu Photonics, http://www.hamamatsu.com/resources/pdf/etd/PMT_handbook_v3aE.pdf

[45] Tan, H. H. (1982) A statistical model of the photomultipliers gain process with applications to optical pulse detection. TDA Prog. Rep. 42-68

[46] Stokey, R. J. and Lee, P. J. (1983) Approximation to the probability density at the output of a photomultipliers tube. TDA Prog. Rep. 42-73
