# Supplementary material for: A Computational Framework for Bioimaging Simulation
Source: PLoS One. 2015 Jul 6;10(7):e0130089. doi: 10.1371/journal.pone.0130089 (PMC4509736; doi:10.1371/journal.pone.0130089)
Supplement: S2 Text — (PDF) [file pone.0130089.s002.pdf]

S2 Text. Details of *in vitro* image comparison

(1) HaloTag-TMR molecules on glass surface

In vitro **Experiment :** HaloTag-TMR molecules were provided by Dr. Masahiro Ueda, laboratory for cell signaling dynamics, RIKEN QBiC. Data was taken by Dr. Satomi Matsuoka, laboratory for cell signaling dynamics, RIKEN QBiC. The molecules were distributed on glass surface, and observed using total internal reflection microscopy with 60X/1.40NA objective (Nikon). Fluorescent images of the HaloTag-TMR molecules are acquired with an EMCCD camera (iXon+, Andor). The images were obtained at a 30 msec exposure time.

Particle model : We constructed simple model of 100 stationary HaloTag tetramethyl rhodamine (TMR) molecules distributed on glass surface.

Simulated imaging : We simulated imaging the basal region of the particle model for the pecification and condition of the TIRFM simulation module shown in Table S2.1.

Beam flux density	20, 30, 40, 50 W/cm^2
Beam wavelength	488nm
Refraction index	1.33 (glass), 1.27 (water)
Critical angle	65.6°
Fluorophore	HaloTag TMR ligand (Abs. 555 nm/ Em. 585 nm)
Objective	× 60 / N.A. 1.40
Dichroic mirror	Semrok FF-562-Di03
Emission filter	Semrok FF-593-25/40
Linear conversion	10^{-6}
Tube lens	× 3.3
Optical magnification	× 198
Camera type	EMCCD (iXon+ Andor)
Image size	512 × 512
Pixel size	16 μm
QE	92 %
EM Gain	× 300
Exposure time	30 msec
Readout noise	100 electrons
Full well	180,000 electrons
Dynamic range	71.1 dB
Excess noise	$\sqrt{2}$
A/D Converter	16-bit
Gain	11.1 electrons/count
Offset	100 counts
Optical background	1.0 photons/pixel

S2 Table S2.1: TIRFM specifications and condition to image the simple particle model of fluorescent molecules.

Expected images **Simulated images**

Beam flux density : 20 W/cm^2

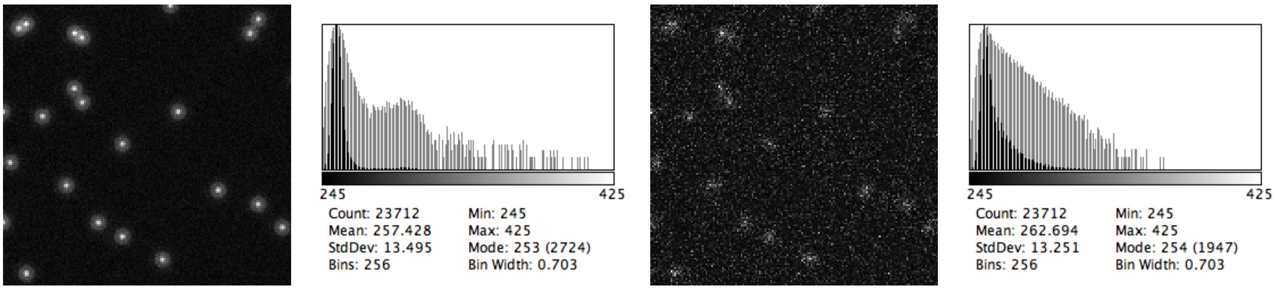

Beam flux density : 30 W/cm^2

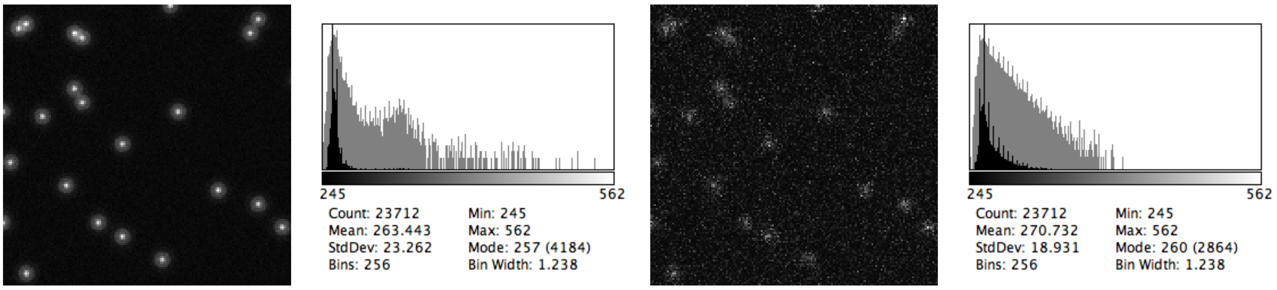

Beam flux density : 40 W/cm^2

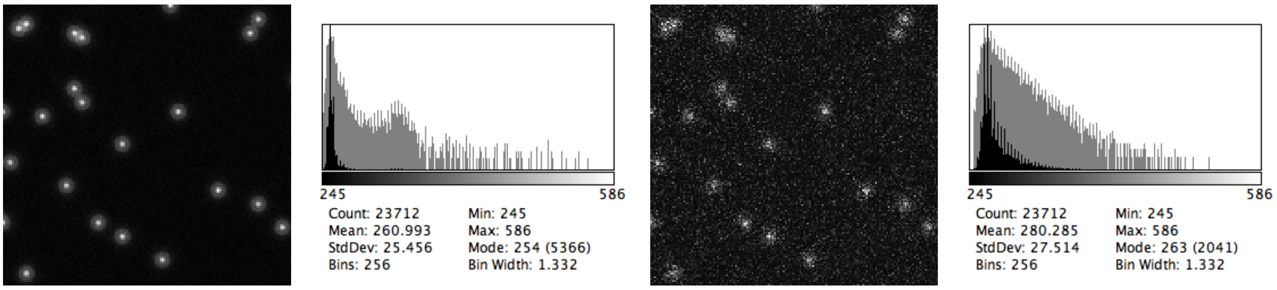

Beam flux density : 50 W/cm^2

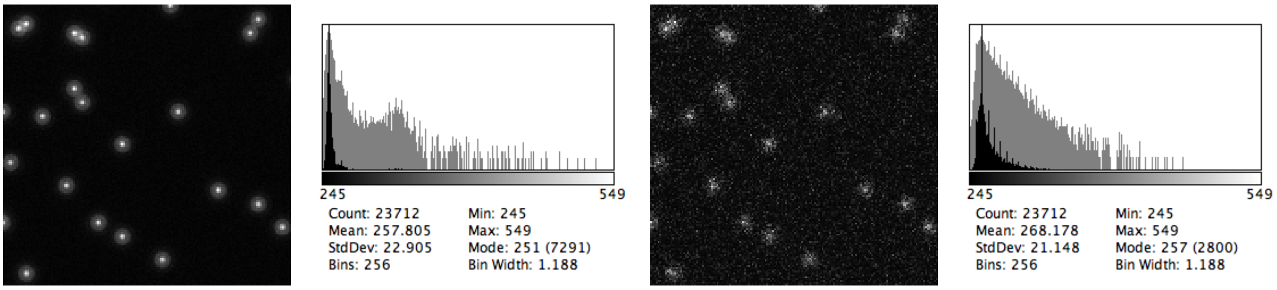

S2 Fig. S2.1: Comparison of *in vitro* images (156 × 152 pixels) and intensity histograms. Log-scaled intensity histograms are shown in grey color.

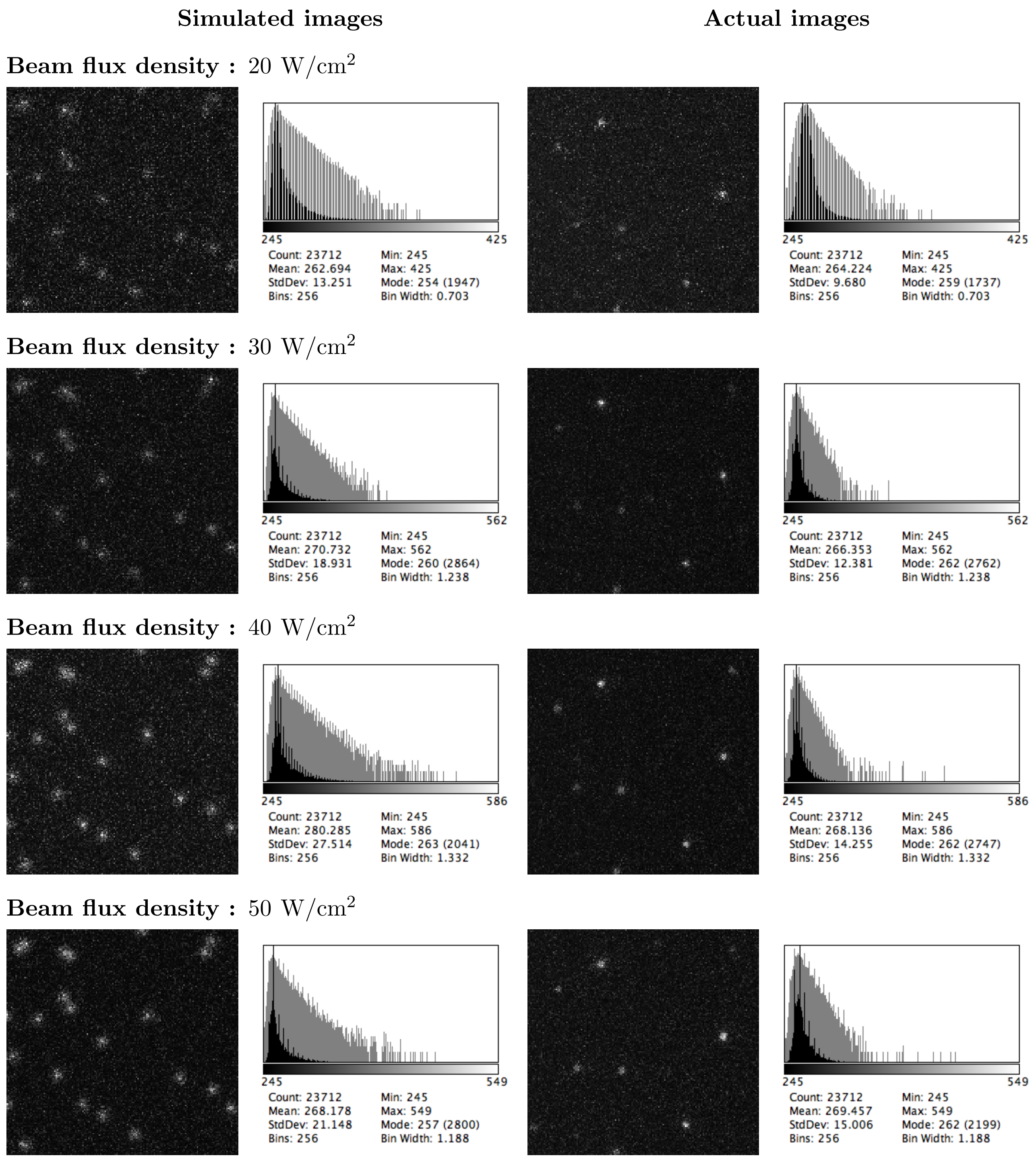

S2 Fig. S2.2: Comparison of *in vitro* images (156 × 152 pixels) and intensity histograms. Log-scaled intensity histograms are shown in grey color.

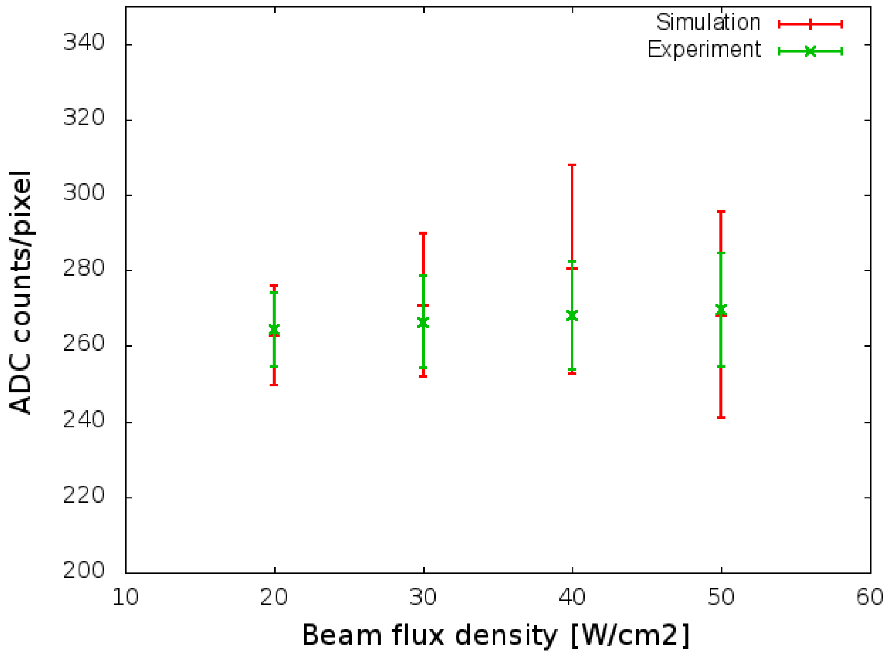

S2 Fig. S2.3: Linearity is shown for various beam flux density. Experiment (green) and simulation (red).

(2) HaloTag-TMR molecules in aqueous solution

In vitro **Experiment :** HaloTag-TMR molecules were provided by Dr. Masahiro Ueda, laboratory for cell signaling dynamics, RIKEN QBiC. Data was taken by Dr. Satomi Matsuoka, laboratory for cell signaling dynamics, RIKEN QBiC. 5 nM concentration of HaloTag-TMR molecules in aqueous solution were observed using a laser scanning confocal microscope (A1; Nikon, Japan) with 60X/1.40NA objective (Nikon). Images of the HaloTag-TMR molecules were obtained at a time resolution of 1 sec.

Particle model : We constructed the particle model of $19,656$ HaloTag-TMR molecules fast diffusing with 100 μm^2/sec and distributed in $30 \times 30 \times 6$ μm^3 box of aqueous solutions.

Simulated imaging : We simulated imaging the middle region of the particle model for the specification and condition of the LSCM simulation module shown in Table S2.2.

Beam flux	5, 10, 30, 50, 100 μW
Beam wavelength	512 nm
Beam waist	400 nm (Assumed)
Fluorophore	HaloTag TMR ligand (Abs. 555 nm/ Em. 585 nm)
Objective	$\times$ 60 / N.A. 1.49
Scan lens	$\times$ 1
Pinhole	57.6 μm diameter (2 A.U)
Optical magnification	$\times$ 60
Linear conversion	10^{-6}
Scan time	0.95 μsec/pixel
Pixel length	207.16 nm/pixel
Image size	1024×1024
PMT mode	Photon-counting
A/D Converter	12-bit
Gain	1.025 electrons/count
Offset	100 counts
Readout noise	0 counts/sec
Excess noise	N/A
Optical background	0 photons

S2 Table S2.2: LSCM specifications and condition to image the simple particle model of fluorescent molecules.

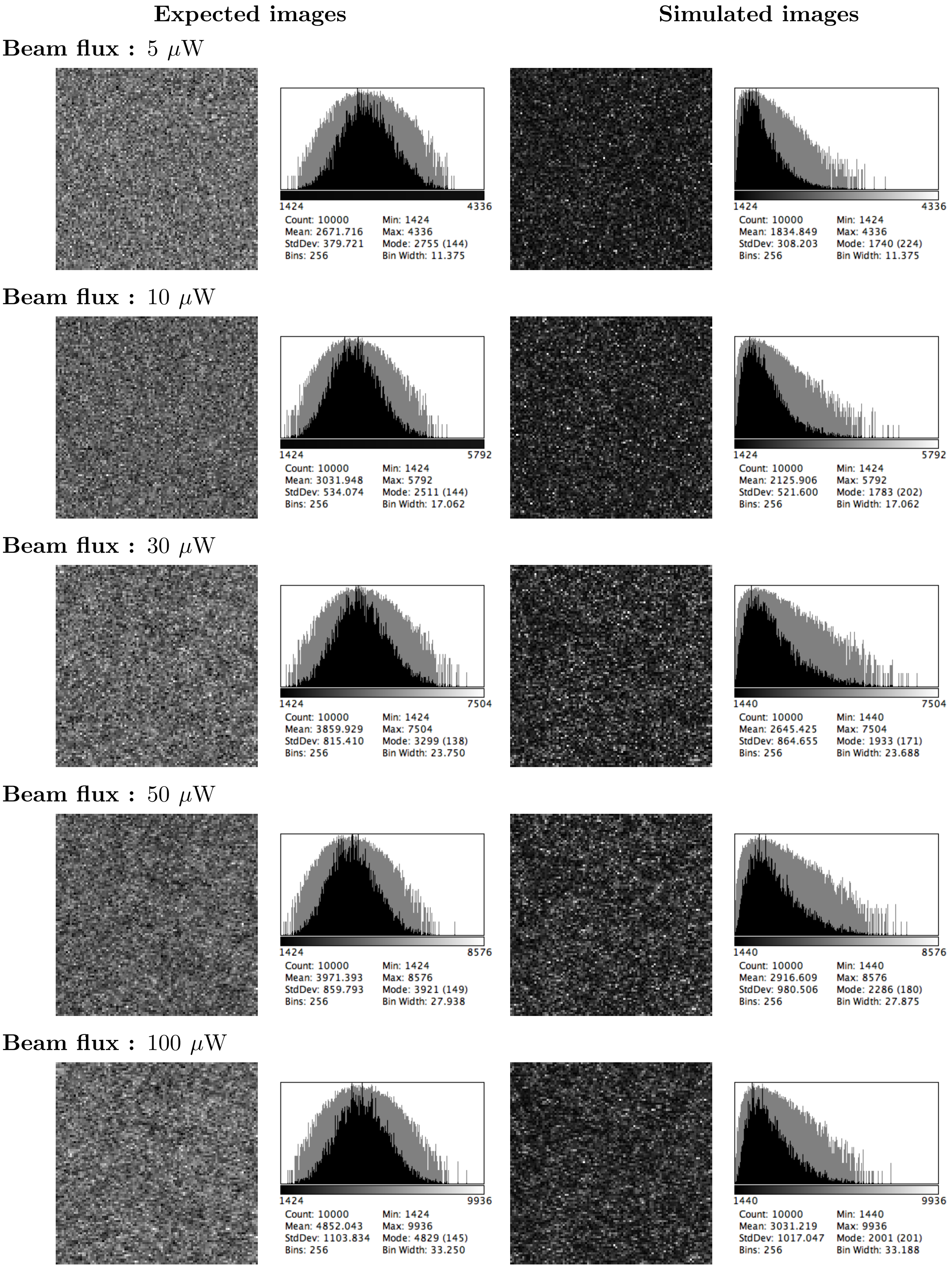

S2 Fig. S2.4: Comparison of *in vitro* images (100 × 100 pixels) and intensity histograms. Log-scaled intensity histograms are shown in grey color. The PMT dark current has not been simulated yet.

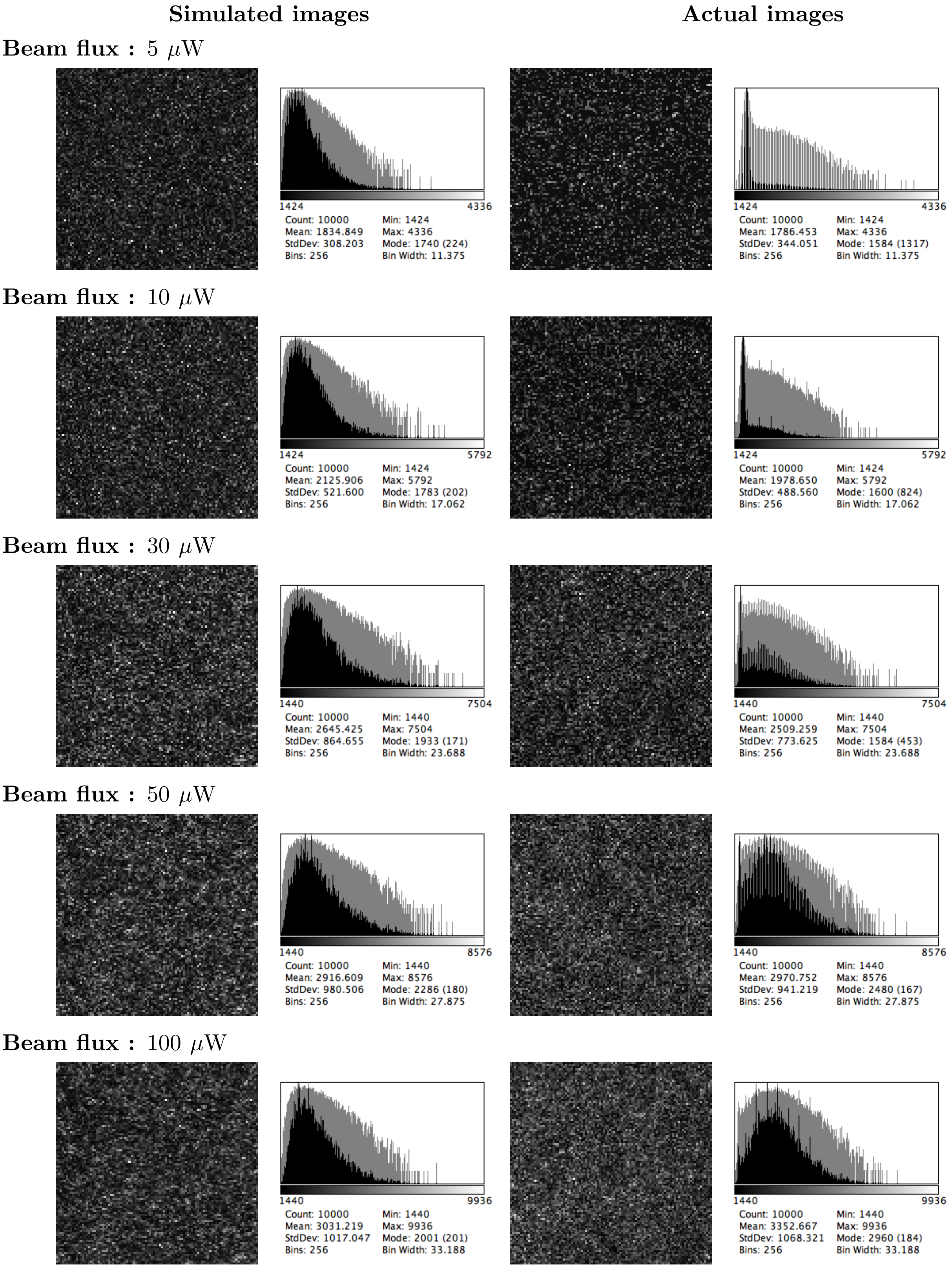

S2 Fig. S2.5: Comparison of *in vitro* images (100×100 pixels) and intensity histograms. Log-scaled intensity histograms are shown in grey color. The PMT dark current has not been simulated yet.

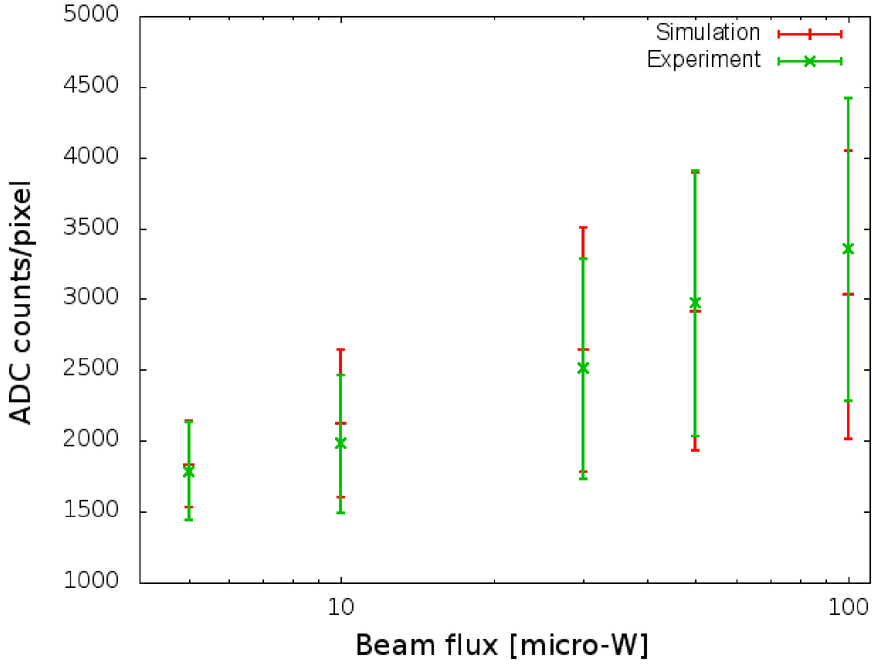

S2 Fig. S2.6: Linearity is shown for various beam flux. Experiment (green) and simulation (red).
