# Supplementary material for: A Computational Framework for Bioimaging Simulation
Source: PLoS One. 2015 Jul 6;10(7):e0130089. doi: 10.1371/journal.pone.0130089 (PMC4509736; doi:10.1371/journal.pone.0130089)
Supplement: S3 Text — (PDF) [file pone.0130089.s003.pdf]

S3 Text. Details of *in vivo* image comparison

(i) ERK nuclear translocation model of EGF signaling pathway

Cell preparation : Rat PC12 pheochromocytoma cells stably expressing mEGFP-tagged ERK2 were provided by Dr. Yasushi Sako, Cellular Informatics Laboratory, RIKEN. Data was taken by Yuki Shindo, laboratory for biochemical simulation, RIKEN QBiC. Cells were plated on poly-L-lysine coated coverslips and cultured for 12 h in Dulbecco's modified Eagle's medium (DMEM) supplemented with 10% hrs serum and 5% fetal bovine serum. Then, cells were serum-starved for 16 h in DMEM without fenol-red supplemented with 1% BSA (DMEM-BSA). Before microcopy experiments, the medium was changed to DMEM-BSA containing 5 mM PIPES (pH 7.2).

Timelapse imaging : mEGFP-ERK2 proteins in living PC12 cells were observed using a laser scanning confocal microscope (A1; Nikon, Japan) with 60X/1.49NA objective (Nikon). Cells were stimulated with epidermal growth facotr (EGF) (5 ng ml^{-1} final concentration) on the microscope at room temperature. Timelapse movies were obtained at a time resolution of 1 min.

Cell model : A particle detailed ERK nuclear translocation model of the EGF signalling pathway is constructed by Dr. Kazunari Iwamoto, laboratory for biochemical simulation, RIKEN QBiC. The model consists of 73 chemical species, 144 reactions and 85 kinetic parameters. The EGF signalling pathway regulates cellular proliferation, differentiation and apoptosis [3]. EGF ligands bind to EGF receptors, which are dimerized and subsequently autophosphorylated. Adaptor proteins, Shc and Grb2, bind to the phosphorylated receptors to form a signalling complex. Sos binds to the signaling complex and then promotes the Ras-GDP/Ras-GTP exchange [4]. Although both Ras-GDP and Ras-GTP bind to Raf protein at cellular membrane, only Ras-GTP can activate Raf [5]. Activated Raf doubly phosphorylates and activates MEK at cytoplasm. Active MEK also doubly phosphorylates ERK, followed by the translocation of phosphorylated ERK from cytoplasm into nucleus [6, 7]. Phosphorylated ERK negatively regulates the signaling complex through the phosphorylation of Sos [8]. We simulated the cell model using the Spatiocyte method. See Text S4 for model parameterisation.

Simulated imaging : We simulated imaging the middle region of the cell model for the specification and condition of the LSCM simulation module shown in Table S3.1.

Beam flux	10 μW (Assumed)
Beam wavelength	488 nm
Beam waist	200 nm (Assumed)
Fluorophore	mEGFP (Abs. 484 nm/ Em. 507 nm)
Objective	$\times$ 60 / N.A. 1.49
Scan lens	$\times$ 1
Pinhole	57.6 μm diameter (2 A.U)
Optical magnification	$\times$ 60
Linear conversion	10^{-6}
Scan time	1.15 μsec/pixel
Pixel length	207.16 nm/pixel
Image size	1024 $\times$ 1024
PMT mode	Analog
A/D Converter	12-bit
QE	30 %
Gain	$\times 10^6$
Dynode	11 stages
Readout noise	0 mA
Excess noise	1.1
Optical background	0 photons/sec

S3 Table S3.1: LSCM specifications and condition to image the ERK nuclear translocation model of EGF signaling pathway.

(ii) Self-organizing wave model for the chemotactic pathway of *D. discoideum*

Cell preparation : *Dictyostelium discoideum* cells were provided by Dr. Masahiro Ueda, laboratory for cell signaling dynamics, RIKEN QBiC. Data was taken by Seiya Fukushima, Graduate School of Frontier Bioscience, Osaka University. Cell preparation and growth conditions were described in ref. [1, 2].

Timelapse imaging : PTEN-TMR and PH-EGFP in living *Dictyostelium discoiduem* cells were observed using a laser scanning confocal microscope (A1; Nikon, Japan) with 60X/1.49NA objective (Nikon). Images of PH-EGFP and PTEN-TMR-expressing cells were obtained at a time resolution of 5 sec.

Cell model : *Dictyostelium discoideum* migrates toward the elevated side of 3'-5'-cyclic adenosine monophosphate (cAMP) external gradient by extending pseudopodia. The accumulation of phosphatidylinositol 3,4,5-trisphosphate (PIP3) lipid and F-actin at the leading edge of the cell is necessary for the pseudopodia formation. When F-actin polymerization is inhibited in the absence of chemoattractant, the cells maintain their disc-like shape without triggering protrusions. Despite the absence of F-actin membrane accumulation, self-organized waves of PIP3 are spontaneously generated on the membrane of these cells. The waves are regulated by phosphatase and tensin homolog (PTEN) and phosphoinositide-3-kinase (PI3K). A detailed particle model of the waves was constructed by Dr. Satya N. V. Arjunan, laboratory for biochemical simulation, RIEKN QBiC. The model consists of 8 chemical species, 12 reactions and 17 kinetic parameters. On the membrane, PI3K phosphorylates phosphatidylinositol 3,4,5-biphosphate (PIP2) into PIP3, whereas PTEN dephosphorylates PIP3 into PIP2. Cytosolic PTEN is recruited to the membrane regions containing PIP2. Nonetheless, PIP3 can dislodge PTEN from PIP2 into the cytosol when it comes in contact. This last reaction acts a positive feedback for PIP3 accumulation. See Text S5 for model parameterisation.

Simulated imaging : We simulated imaging the middle region of the cell model for the specification and condition of the LSCM simulation module are shown in Table S3.2.

Beam flux 1	10 μW (Assumed)
Beam wavelength 1	488 nm
Beam waist 1	200 nm (Assumed)
Fluorophore 1	EGFP (Abs. 384 nm/ Em. 509 nm)
Beam flux 2	10 μW
Beam wavelength 2	561 nm
Beam waist 2	200 nm (Assumed)
Fluorophore 2	TRITC (Abs. 584 nm/ Em. 608 nm)
Objective	$\times$ 60 / N.A. 1.49
Scan lens	$\times$ 1
Pinhole	37 μm diameter (2 A.U)
Optical magnification	$\times$ 60
Linear conversion	10^{-6}
Scan time	4.27 μsec/pixel
Pixel length	414.3 nm/pixel
Image size	512 $\times$ 512
Detector	PMT : Analog mode
A/D Converter	12-bit
QE	30 %
Readout noise	0 mA
Gain	$\times 10^6$
Dynode	11 stages
Excess noise	1.1
Optical background	0.00 photons/pixel

S3 Table S3.2: 2-color LSCM specifications and condition to image the self-organizing wave model of *Dictyostelium discoiduem* cell.

References

[1] Arai,Y. *et al.* (2010) Self-organization of the phosphatidylinositol lipids signaling system for random cell migration. Proc. Natl. Acad. Sci. U. S. A., 107, 12399-404.

[2] Shibata, T. et al. (2012) Modeling the self-organized phosphatidylinositol lipid signaling system in chemotactic cells using quantitative image analysis. J. Cell Sci., 125, 5138-50.

[3] Yarden, Y., and Sliwkowski, M. X. (2001) Untangling the ErbB signalling network. Nat Rev Mol Cell Biol 2: 127-137.

[4] Corbalan-Garcia, S., Margarit, S. M., Galron, D., Yang, S. S. and Baar-Sag, D. (1998) Regulation of Sos activity by intramolecular interactions. Mol. Cell. Biol. 18, 880-886.

[5] Hibino, K., Shibata, T., Yanagida, T., and Sako, Y. Activation kinetics of RAF protein in the Ternary Complex of RAF, RAS-GTP, and kinase on the plasma membrane of living cells. *J. Biol. Chem.*, 2011, 286, 36460-36468.

[6] Fujioka, A. *et al.* "Dynamics of the Ras/ERK MAPK cascade as monitored by fluorescent probes." Journal of biological chemistry 281.13 (2006): 8917-8926.

[7] Cohen-Saidon, C., *et al.* "Dynamics and variability of ERK2 response to EGF in individual living cells." Molecular cell 36.5 (2009): 885-893.

[8] Sturm, Oliver E., *et al.* "The mammalian MAPK/ERK pathway exhibits properties of a negative feedback amplifier." *Science signaling* 3.153 (2010): ra90.
