# Supplementary material for: A Computational Framework for Bioimaging Simulation
Source: PLoS One. 2015 Jul 6;10(7):e0130089. doi: 10.1371/journal.pone.0130089 (PMC4509736; doi:10.1371/journal.pone.0130089)
Supplement: S4 Text — (PDF) [file pone.0130089.s004.pdf]

**S4 Text. Parameterization for ERK nuclear translocation model**				
Simuation setting				
Voxel radius	2e-8 m			
Shape	Half egg			
Size	20microm, 20microm, 10microm			
Compartments	Membrane, cytoplasm, nucleus			
Cytoplasmic volmicrome	9.53e-16 m			
Nucleus volmicrome	2.97e-16 m			

No	Name	Molecules	Diffusion cofficient	Description	Location
1	EGFR	20000	0.015 microm2/s	EGF receptor	Membrane
2	EGFR_EGF	0	0.015 microm2/s	EGF-bind EGFR	Membrane
3	dEGFR	0	0.015 microm2/s	Dimerized EGFR	Membrane
4	dEGFRP	0	0.015 microm2/s	Phosphorylated dimer of EGFR	Membrane
5	dEGFRP_GAP	0	0.015 microm2/s		Membrane
6	dEGFRP_GAP_Grb2	0	0.015 microm2/s		Membrane
7	dEGFRP_GAP_Grb2_Sos	0	0.015 microm2/s	Active form of EGFR	Membrane
8	dEGFRP_GAP_Grb2_Sos_RasGDP	0	0.015 microm2/s		Membrane
9	dEGFRP_GAP_Grb2_Sos_dRasGTP	0	0.015 microm2/s		Membrane
10	dEGFRP_GAP_Grb2_Sos_ERKPP	0	0.015 microm2/s		Membrane
11	deg_dEGFR_GAP_Grb2_Sos	0	0.015 microm2/s	Degradation form of active EGFR	Membrane
12	dEGFRP_GAP_Shc	0	0.015 microm2/s		Membrane
13	dEGFRP_GAP_ShcP	0	0.015 microm2/s		Membrane
14	dEGFRP_GAP_ShcP_Grb2	0	0.015 microm2/s		Membrane
15	dEGFRP_GAP_ShcP_Grb2_Sos	0	0.015 microm2/s	Active form of EGFR	Membrane
16	dEGFRP_GAP_ShcP_Grb2_Sos_RasGDP	0	0.015 microm2/s		Membrane
17	dEGFRP_GAP_ShcP_Grb2_Sos_dRasGTP	0	0.015 microm2/s		Membrane
18	dEGFRP_GAP_ShcP_Grb2_Sos_ERKPP	0	0.015 microm2/s		Membrane
19	deg_dEGFRP_GAP_ShcP_Grb2_Sos	0	0.015 microm2/s	Degradation form of active EGFR	Membrane
20	RasGDP	30000	0.015 microm2/s		Membrane
21	RasGTP	0	0.015 microm2/s	Active form of RasGTP	Membrane
22	dRasGTP	0	0.015 microm2/s	Inactive form of RasGTP	Membrane
23	Raf_RasGDP	0	0.015 microm2/s		Membrane
24	Raf_RasGTP	0	0.015 microm2/s		Membrane
25	Inter_Raf_RasGTP	0	0.015 microm2/s	Intermediate of Raf_RasGTP complex	Membrane
26	EGFRi	0	1 microm2/s	Internalized EGFR	Cytoplasm
27	EGFRi_EGF	0	1 microm2/s	Internalized EGFRi_EGF	Cytoplasm
28	dEGFRi	0	1 microm2/s	Internalized EGFRi	Cytoplasm
29	dEGFRPi	0	1 microm2/s	Internalized EGFRPi	Cytoplasm
30	dEGFR_cyto	0	1 microm2/s	Degradation form of internalized EGFRs	Cytoplasm
31	EGFi	0	1 microm2/s	Internalized EGF	Cytoplasm
32	GAP	1500	1 microm2/s		Cytoplasm
33	Grb2	1250	1 microm2/s		Cytoplasm
34	Sos	3250	1 microm2/s		Cytoplasm
35	Grb2_Sos	5000	1 microm2/s		Cytoplasm
36	Shc	13750	1 microm2/s		Cytoplasm
37	ShcP	0	1 microm2/s		Cytoplasm
38	ShcP_Grb2_Sos	0	1 microm2/s		Cytoplasm
39	ShcP_Grb2	0	1 microm2/s		Cytoplasm
40	Raf	1000	1 microm2/s		Cytoplasm
41	Pase1	250	1 microm2/s		Cytoplasm
42	ActRaf	0	1 microm2/s		Cytoplasm
43	ActRaf_Pase1	0	1 microm2/s		Cytoplasm
44	MEK_ActRaf	0	1 microm2/s		Cytoplasm
45	MEKP_ActRaf	0	1 microm2/s		Cytoplasm
46	MEK	97860	1 microm2/s		Cytoplasm
47	MEKP	0	1 microm2/s		Cytoplasm
48	MEKPP	0	1 microm2/s		Cytoplasm
49	MEKPP_Pase2	0	1 microm2/s		Cytoplasm
50	MEKP_Pase2	0	1 microm2/s		Cytoplasm
51	Pase2	19185	1 microm2/s		Cytoplasm
52	ERK	57000	1 microm2/s		Cytoplasm
53	ERK_MEKPP	0	1 microm2/s		Cytoplasm
54	ERKP	0	1 microm2/s		Cytoplasm
55	ERKP_MEKPP	0	1 microm2/s		Cytoplasm
56	ERKPP	0	1 microm2/s		Cytoplasm
57	Pase3	1918	1 microm2/s		Cytoplasm
58	ERKPP_Pase3	0	1 microm2/s		Cytoplasm
59	ERKP_Pase3	0	1 microm2/s		Cytoplasm
60	ERKPP_Sos	0	1 microm2/s		Cytoplasm
61	iSos	0	1 microm2/s	Inactive form of Sos	Cytoplasm
62	nERK	18000	1 microm2/s		Nucleus
63	nERKP	0	1 microm2/s		Nucleus
64	nERKPP	0	1 microm2/s		Nucleus
65	nPase3	581	1 microm2/s		Nucleus
66	nERKPP_Pase3	0	1 microm2/s		Nucleus
67	nERKP_Pase3	0	1 microm2/s		Nucleus
68	nMEK	7140	1 microm2/s		Nucleus
69	nMEKP	0	1 microm2/s		Nucleus
70	nMEKPP	0	1 microm2/s		Nucleus
71	nERK_MEKPP	0	1 microm2/s		Nucleus
72	nERKP_MEKPP	0	1 microm2/s		Nucleus
73	nPase2	5815	1 microm2/s		Nucleus
74	nMEKPP_Pase2	0	1 microm2/s		Nucleus
75	nMEKP_Pase2	0	1 microm2/s		Nucleus

EGFR activation module

No.	Reaction	Value	Unit
1	EGFR -> EGFR_EGF	4e7*EGFconc(M)	/M/s
2	EGFR_EGF -> EGFR	0.04	/s
3	EGFR_EGF + EGFR_EGF -> dEGFR	3.45E-14	m
4	dEGFR -> EGFR_EGF + EGFR_EGF	0.1	/s
5	dEGFR -> dEGFRP	0.05	/s
6	dEGFRP -> dEGFR	0.025	/s
7	dEGFRP + GAP -> dEGFRP_GAP	8.50E-21	m
8	dEGFRP_GAP -> dEGFRP + GAP	0.2	/s
9	dEGFRP_GAP + Shc -> dEGFRP_GAP_Shc	3.07E-20	m
10	dEGFRP_GAP_Shc -> dEGFRP_GAP + Shc	0.1	/s
11	dEGFRP_GAP_Shc -> dEGFRP_GAP_ShcP	0.1	/s
12	dEGFRP_GAP_ShcP -> dEGFRP_GAP_Shc	0.006	/s
13	dEGFRP_GAP + Grb2 -> dEGFRP_GAP_Grb2	1.56E-20	m
14	dEGFRP_GAP_Grb2 -> dEGFRP_GAP + Grb2	0.275	/s
15	dEGFRP_GAP_ShcP + Grb2 -> dEGFRP_GAP_ShcP_Grb2	1.56E-20	m
16	dEGFRP_GAP_ShcP_Grb2 -> dEGFRP_GAP_ShcP + Grb2	0.55	/s
17	dEGFRP_GAP_Grb2 + Sos -> dEGFRP_GAP_Grb2_Sos	1.56E-20	m
18	dEGFRP_GAP_Grb2_Sos -> dEGFRP_GAP_Grb2 + Sos	0.06	/s
19	dEGFRP_GAP_ShcP_Grb2 + Sos -> dEGFRP_GAP_ShcP_Grb2_Sos	1.56E-20	m
20	dEGFRP_GAP_ShcP_Grb2_Sos -> dEGFRP_GAP_ShcP_Grb2 + Sos	0.0214	/s
21	dEGFRP_GAP_Grb2_Sos + RasGDP -> dEGFRP_GAP_Grb2_Sos_RasGDP	5.00E-14	m
22	dEGFRP_GAP_Grb2_Sos_RasGDP -> dEGFRP_GAP_Grb2_Sos + RasGDP	1.3	/s
23	dEGFRP_GAP_Grb2_Sos_RasGDP -> dEGFRP_GAP_Grb2_Sos + RasGTP	0.5	/s
24	dEGFRP_GAP_ShcP_Grb2_Sos + RasGDP -> dEGFRP_GAP_ShcP_Grb2_Sos_RasGDP	5.00E-14	m
25	dEGFRP_GAP_ShcP_Grb2_Sos_RasGDP -> dEGFRP_GAP_ShcP_Grb2_Sos + RasGDP	1.3	/s
26	dEGFRP_GAP_ShcP_Grb2_Sos_RasGDP -> dEGFRP_GAP_ShcP_Grb2_Sos + RasGTP	0.5	/s
27	dEGFRP_GAP_Grb2_Sos + dRasGTP -> dEGFRP_GAP_Grb2_Sos_dRasGTP	1.00E-14	m
28	dEGFRP_GAP_Grb2_Sos_dRasGTP -> dEGFRP_GAP_Grb2_Sos + dRasGTP	0.4	/s
29	dEGFRP_GAP_Grb2_Sos_dRasGTP -> dEGFRP_GAP_Grb2_Sos + RasGDP	0.023	/s
30	dEGFRP_GAP_ShcP_Grb2_Sos + dRasGTP -> dEGFRP_GAP_ShcP_Grb2_Sos_dRasGTP	1.00E-14	m
31	dEGFRP_GAP_ShcP_Grb2_Sos_dRasGTP -> dEGFRP_GAP_ShcP_Grb2_Sos + dRasGTP	0.4	/s
32	dEGFRP_GAP_ShcP_Grb2_Sos_dRasGTP -> dEGFRP_GAP_ShcP_Grb2_Sos + RasGDP	0.023	/s
33	RasGDP + Raf -> Raf_RasGDP	1.56E-24	m
34	Raf_RasGDP -> RasGDP + Raf	3.7	/s
35	RasGTP + Raf -> Raf_RasGTP	1.56E-20	m
36	Raf_RasGTP -> RasGTP + Raf	0.04	/s
37	Raf_RasGTP -> Inter_Raf_RasGTP	0.8	/s
38	Inter_Raf_RasGTP -> dRasGTP + ActRaf	2.4	/s
39	EGFRi + VACANT -> EGFR	9.62E-21	m
40	EGFR -> EGFRi	0.001	/s
41	dEGFRP -> dEGFRPi	0.001	/s
42	dEGFRPi + VACANT -> dEGFRP	9.62E-21	m
43	dEGFRP_GAP -> dEGFR_cyto	0.001	/s
44	dEGFRP_GAP_Grb2 -> dEGFR_cyto	0.001	/s
45	dEGFRP_GAP_Grb2_Sos -> dEGFR_cyto	0.001	/s
46	dEGFRP_GAP_Shc -> dEGFR_cyto	0.001	/s
47	dEGFRP_GAP_ShcP -> dEGFR_cyto	0.001	/s
48	dEGFRP_GAP_ShcP_Grb2 -> dEGFR_cyto	0.001	/s
49	dEGFRP_GAP_ShcP_Grb2_Sos -> dEGFR_cyto	0.001	/s
50	dEGFRP_GAP + ShcP -> dEGFRP_GAP_ShcP	1.49E-21	m
51	dEGFRP_GAP_ShcP -> dEGFRP_GAP + ShcP	0.3	/s
52	dEGFRP_GAP + ShcP_Grb2 -> dEGFRP_GAP_ShcP_Grb2	1.49E-21	m
53	dEGFRP_GAP_ShcP_Grb2 -> dEGFRP_GAP + ShcP_Grb2	0.3	/s
54	dEGFRP_GAP + ShcP_Grb2_Sos -> dEGFRP_GAP_ShcP_Grb2_Sos	3.99E-22	m
55	dEGFRP_GAP_ShcP_Grb2_Sos -> dEGFRP_GAP + ShcP_Grb2_Sos	0.1	/s
56	dEGFRP_GAP + Grb2_Sos -> dEGFRP_GAP_Grb2_Sos	7.28E-21	m
57	dEGFRP_GAP_Grb2_Sos -> dEGFRP_GAP + Grb2_Sos	0.03	/s
58	dEGFRP_GAP_ShcP + Grb2_Sos -> dEGFRP_GAP_ShcP_Grb2_Sos	7.28E-21	m
59	dEGFRP_GAP_ShcP_Grb2_Sos -> dEGFRP_GAP_ShcP + Grb2_Sos	0.03	/s
60	dEGFRP_GAP_Grb2_Sos + ERKPP -> dEGFRP_GAP_Grb2_Sos_ERKPP	1.56E-20	m
61	dEGFRP_GAP_Grb2_Sos_ERKPP -> dEGFRP_GAP_Grb2_Sos + ERKPP	0.01	/s
62	dEGFRP_GAP_Grb2_Sos_ERKPP -> deg_dEGFRP_GAP_Grb2_Sos + ERKPP	0.2	/s
63	dEGFRP_GAP_ShcP_Grb2_Sos + ERKPP -> dEGFRP_GAP_ShcP_Grb2_Sos_ERKPP	1.56E-20	m
64	dEGFRP_GAP_ShcP_Grb2_Sos_ERKPP -> dEGFRP_GAP_ShcP_Grb2_Sos + ERKPP	0.01	/s
65	dEGFRP_GAP_ShcP_Grb2_Sos_ERKPP -> deg_dEGFRP_GAP_ShcP_Grb2_Sos + ERKPP	0.2	/s
66	EGFRi -> VACANT	0.0055	/s
67	EGFRi_EGFi + EGFRi_EGFi -> dEGFRi	1.56E-20	m
68	dEGFRi -> EGFRi_EGFi + EGFRi_EGFi	0.1	/s
69	dEGFRi -> dEGFRPi	1	/s
70	dEGFRPi -> dEGFRi	0.01	/s
71	Grb2 + Sos -> Grb2_Sos	7.28E-21	m
72	Grb2_Sos -> Grb2 + Sos	0.0015	/s
73	ShcP -> Shc	0.005	/s
74	Grb2 + ShcP -> ShcP_Grb2	1.56E-20	m
75	ShcP_Grb2 -> Grb2 + ShcP	0.55	/s
76	Sos + ShcP_Grb2 -> ShcP_Grb2_Sos	4.17E-20	m
77	ShcP_Grb2_Sos -> Sos + ShcP_Grb2	0.062	/s
78	Grb2_Sos + ShcP -> ShcP_Grb2_Sos	3.07E-20	m
79	ShcP_Grb2_Sos -> Grb2_Sos + ShcP	0.2	/s

Cytoplasmic Raf/MEK/ERK module

No	Reaction	Value	Unit
80	ActRaf + Pase1 -> ActRaf_Pase1	2.07E-19	m
81	ActRaf_Pase1 -> ActRaf + Pase1	2	/s
82	ActRaf_Pase1 -> Raf + Pase1	50	/s
83	MEK + ActRaf -> MEK_ActRaf	1.10E-19	m
84	MEK_ActRaf -> MEK + ActRaf	0.33	/s
85	MEK_ActRaf -> MEKP + ActRaf	35	/s
86	MEKP + ActRaf -> MEKP_ActRaf	1.10E-19	m
87	MEKP_ActRaf -> MEKP + ActRaf	0.33	/s
88	MEKP_ActRaf -> MEKPP + ActRaf	29	/s
89	MEKPP + Pase2 -> MEKPP_Pase2	1.22E-19	m
90	MEKPP_Pase2 -> MEKPP + Pase2	8	/s
91	MEKPP_Pase2 -> Pase2 + MEKP	0.568	/s
92	MEKP + Pase2 -> MEKP_Pase2	4.42E-21	m
93	MEKP_Pase2 -> MEKP + Pase2	5	/s
94	MEKP_Pase2 -> Pase2 + MEK	0.568	/s
95	ERK + MEKPP -> ERK_MEKPP	6.58E-20	m
96	ERK_MEKPP -> ERK + MEKPP	0.183	/s
97	ERK_MEKPP -> ERKP + MEKPP	57	/s
98	ERKP + MEKPP -> ERKP_MEKPP	6.58E-20	m
99	ERKP_MEKPP -> ERKP + MEKPP	0.183	/s
100	ERKP_MEKPP -> ERKPP + MEKPP	160	/s
101	ERKPP + Pase3 -> ERKPP_Pase3	1.21E-19	m
102	ERKPP_Pase3 -> ERKPP + Pase3	6	/s
103	ERKPP_Pase3 -> ERKP + Pase3	2.46	/s
104	ERKP + Pase3 -> ERKP_Pase3	6.26E-20	m
105	ERKP_Pase3 -> ERKP + Pase3	5	/s
106	ERKP_Pase3 -> ERK + Pase3	2.46	/s
107	ERKPP + Sos -> ERKPP_Sos	1.00E-19	m
108	ERKPP_Sos -> ERKPP + Sos	0.01	/s
109	ERKPP_Sos -> ERKPP + iSos	0.2	/s

Nuclear Raf/MEK/ERK module

No	Reaction	Value	Unit
110	nMEKPP + nPase2 -> nMEKPP_Pase2	1.22E-19	m
111	nMEKPP_Pase2 -> nMEKPP + nPase2	8	/s
112	nMEKPP_Pase2 -> nPase2 + nMEKP	0.568	/s
113	nMEKP + nPase2 -> nMEKP_Pase2	4.42E-21	m
114	nMEKP_Pase2 -> nMEKP + nPase2	5	/s
115	nMEKP_Pase2 -> nPase2 + nMEK	0.568	/s
116	nERK + nMEKPP -> nERK_MEKPP	6.58E-20	m
117	nERK_MEKPP -> nERK + nMEKPP	0.183	/s
118	nERK_MEKPP -> nERKP + nMEKPP	57	/s
119	nERKP + nMEKPP -> nERKP_MEKPP	6.58E-20	m
120	nERKP_MEKPP -> nERKP + nMEKPP	0.183	/s
121	nERKP_MEKPP -> nERKPP + nMEKPP	160	/s
122	nERKPP + nPase3 -> nERKPP_Pase3	1.21E-19	m
123	nERKPP_Pase3 -> nERKPP + nPase3	6	/s
124	nERKPP_Pase3 -> nERKP + nPase3	2.46	/s
125	nERKP + nPase3 -> nERKP_Pase3	6.26E-20	m
126	nERKP_Pase3 -> nERKP + nPase3	5	/s
127	nERKP_Pase3 -> nERK + nPase3	2.46	/s

Transport between nucleus and cytoplasm

No	Reaction	Value	Unit
128	nERK -> ERK	0.195	/s
129	ERK -> nERK	0.129	/s
130	nERKP -> ERKP	0.2457	/s
131	ERKP -> nERKP	0.3462	/s
132	nERKPP -> ERKPP	0.2457	/s
133	ERKPP -> nERKPP	0.3462	/s
134	nMEK -> MEK	9.15	/s
135	MEK -> nMEK	1.38	/s
136	nMEKP -> MEKP	8.1	/s
137	MEKP -> nMEKP	1.2	/s
138	nMEKPP -> MEKPP	8.1	/s
139	MEKPP -> nMEKPP	1.2	/s
140	nERK_MEKPP -> ERK_MEKPP	3.9	/s
141	ERK_MEKPP -> nERK_MEKPP	1.05	/s
142	nERKP_MEKPP -> ERKP_MEKPP	3.9	/s
143	ERKP_MEKPP -> nERKP_MEKPP	1.05	/s
