# Supplementary material for: A Computational Framework for Bioimaging Simulation
Source: PLoS One. 2015 Jul 6;10(7):e0130089. doi: 10.1371/journal.pone.0130089 (PMC4509736; doi:10.1371/journal.pone.0130089)
Supplement: S5 Text — (PDF) [file pone.0130089.s005.pdf]

**S5 Text. Parameterization for self-organizing wave model of PTEN**				
Simuation setting				
Voxel radius	6e-8 m			
Shape	Half egg			
Size	25microm, 25microm, 13microm			
Compartments	Membrane, cytoplasm			
Cytoplasmic volmicrome	9.53e-16 m			
Nucleus volmicrome	N/A			

No	Name	Molecules	Diffusion cofficient	Description	Location
1	PIP2m	1233	0.01 microm/s	phosphatidylinositol 3,4,5-biphosphate	Membrane
2	PIP3m	0	0.01 microm/s	phosphatidylinositol 3,4,5-trisphosphate	Membrane
3	PIP3a	0	0.01 microm/s	Activated PIP3	Membrane
4	PTENm	309	0.01 microm/s	phosphatase and tensin homolog	Membrane
5	PI3Km	3096	0.01 microm/s	phosphoinositide-3-kinase	Membrane
6	PIP2	8054	N/A	Homogeneously Distributed	Cytoplasm
7	PI3K	9264	N/A	Homogeneously Distributed	Cytoplasm
8	PTEN	6194	N/A	Homogeneously Distributed	Cytoplasm

No.	Reaction	Value	Unit
1	PIP2 -> PIP2m	4E-02	/s
2	PTEN + PIP2m -> PTENm + PIP2m	2E-14	/s
3	PIP3a + PI3K -> PIP3m + PI3Km	1.00E-13	/s
4	PIP3m + PIP3m -> PIP3a + PIP3a	2.24E-14	m
5	PIP2m + PI3Km -> PIP3m + PI3Km	1.17E-14	m
6	PIP3m + PTENm -> PIP2m + PTENm	6.90E-14	m
7	PIP3a + PTENm -> PIP2m + PTENm	6.90E-14	m
8	PTENm -> PTEN	0.09	/s
9	PI3Km -> PI3K	2.00E-02	/s
10	PIP3m -> PIP2	0.02	/s
11	PIP3a -> PIP2	0.02	/s
12	PIP2m -> PIP2	0.001	/s
